# Supplementary material for: Fibroblastic Reticular Cells Control Conduit Matrix Deposition during Lymph Node Expansion
Source: Cell Rep. 2019 Nov 26;29(9):2810–2822.e5. doi: 10.1016/j.celrep.2019.10.103 (PMC6899512; doi:10.1016/j.celrep.2019.10.103)
Supplement: Document S1. Figures S1–S5 [file mmc1.pdf]

Cell Reports, Volume *29*

Supplemental Information

Fibroblastic Reticular Cells Control Conduit Matrix Deposition during Lymph Node Expansion

Victor G. Martinez, Valeriya Pankova, Lukas Krasny, Tanya Singh, Spyridon Makris, Ian J. White, Agnesska C. Benjamin, Simone Dertschnig, Harry L. Horsnell, Janos Kriston-Vizi, Jemima J. Burden, Paul H. Huang, Christopher J. Tape, and Sophie E. Acton

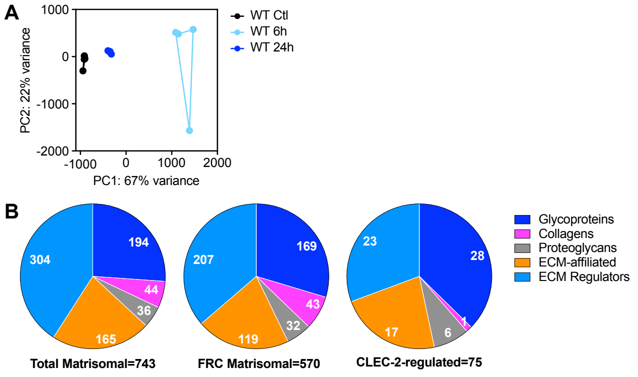

Figure S1. Matrisomal genes regulated by CLEC-2. Gene expression by RNAseq in control FRCs treated with CLEC-2-Fc for 6 and 24 hours. A) CLEC-2-Fc-regulated gene (more or equal than 2-fold) cluster in a PCA space. B) Number of genes per category of matrisomal components in the indicated datasets. Related to Figure 2.

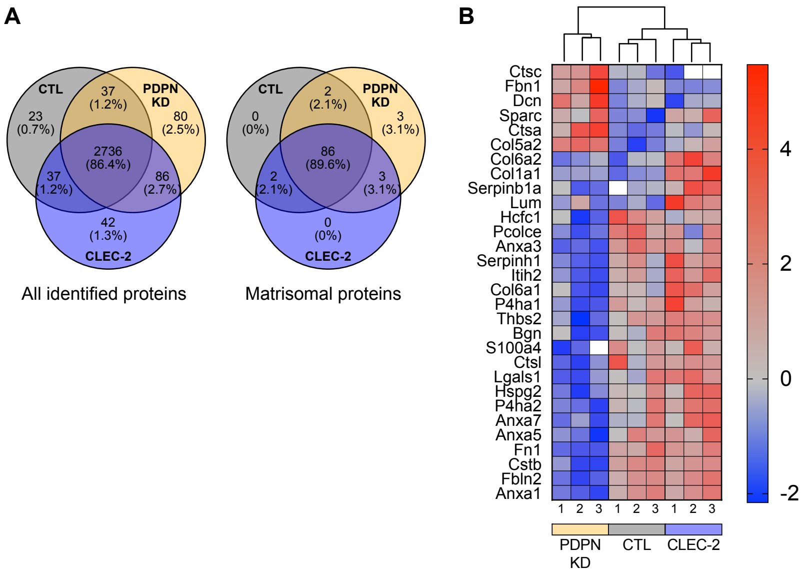

Figure S2. Proteomics of FRC-derived matrices. In vitro FRC cell line-derived matrices generated after 5 days in culture were subjected to proteomic analysis by mass spectrometry. A) Venn diagrams showing overlap between cell lines of proteins detected. Areas shown are not proportional to percentage of overlap. C) Heatmap of matrisomal proteins significantly changed by one-way ANOVA, Tukey's multiple comparisons test. Three replicates for each condition automatically clustered are shown. Colour code represents z-scores. Not detected is represented by the white squares. Related to Figure 4.

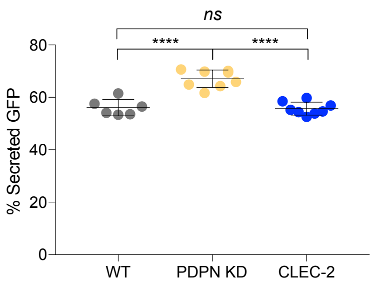

Figure S3. Secretory activity in FRC cell lines. FRC cell lines were transfected to express GFP tagged for secretion. GFP levels in cell supernatants and lysates were determined by ELISA. Dot plot shows percentage of GFP in supernatant relative to total. Dots represent replicates form independent experiments (n=2). ****P<0.00005, one-way ANOVA, Tukey's multiple comparisons test. NS, not significant. Error bars represent mean and SD. Related to Figure 4.

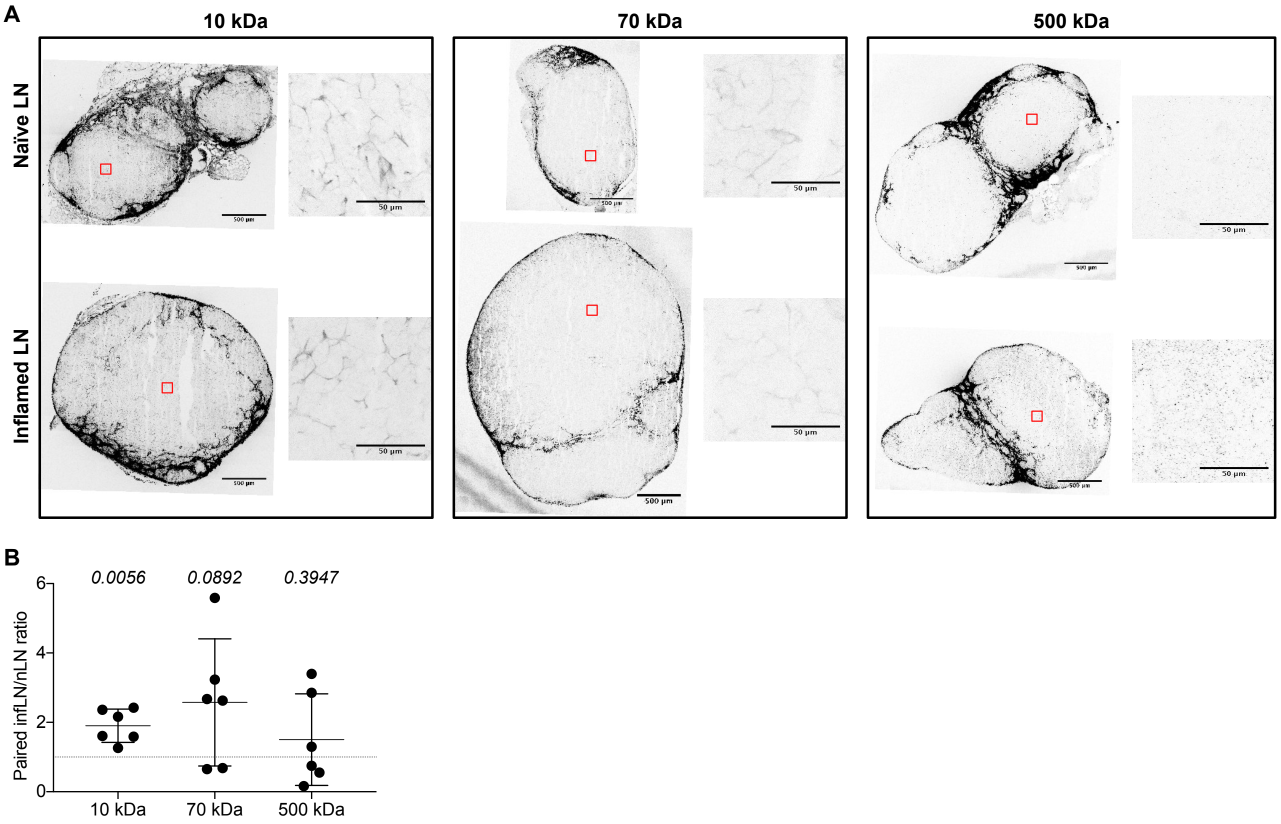

Figure S4. Antigen uptake in vivo. Mice were immunized by subcutaneous injection of IFA/OVA on the right flank. 5 days later, fluorescently labelled dextrans with the indicated sizes were injected on both flanks. A) Immunofluorescence of 20 microns thick cryosections of naïve and inflamed draining LNs 30 minutes post dextran injection. Maximum Z stack projections are shown of representative tile scans and zoomed areas are shown. B) Number of dextran-positive cells per draining LNs 90 minutes after dextran injection was determined by flow cytometry. Dot plot shows ratios between paired inflamed and naïve LNs from same individuals (n=6). Error bars represent mean and SD. P-values are shown for each dextran, unpaired t test. Related to Figure 6.

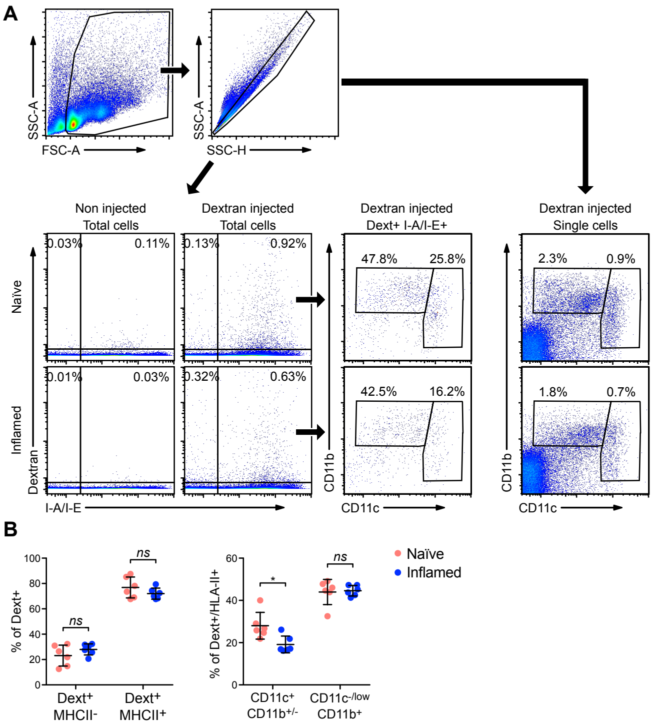

Figure S5. Gating strategy and population analysis for in vivo antigen uptake. A) Representative dot plots showing gating strategy. Live cells were gated according to FSC/SSC parameters and doublets were excluded prior to analysis. Total cells were used to gate dendritic cell (DCs) and macrophage (MF) populations. B) Percentage of the indicated cell populations within the dextran-positive subset. Each dot represents an individual mouse (n=6). Error bars represent mean and SD. *P<0.05, one-way ANOVA, Tukey's multiple comparisons test. Related to Figure 6.
