# Supplementary material for: Fibroblastic Reticular Cells Control Conduit Matrix Deposition during Lymph Node Expansion
Source: Cell Rep. 2019 Nov 26;29(9):2810–2822.e5. doi: 10.1016/j.celrep.2019.10.103 (PMC6899512; doi:10.1016/j.celrep.2019.10.103)
Supplement: Document S2. Article plus Supplemental Information [file mmc3.pdf]

Cell Reports

Article

Fibroblastic Reticular Cells Control Conduit Matrix Deposition during Lymph Node Expansion

Graphical Abstract

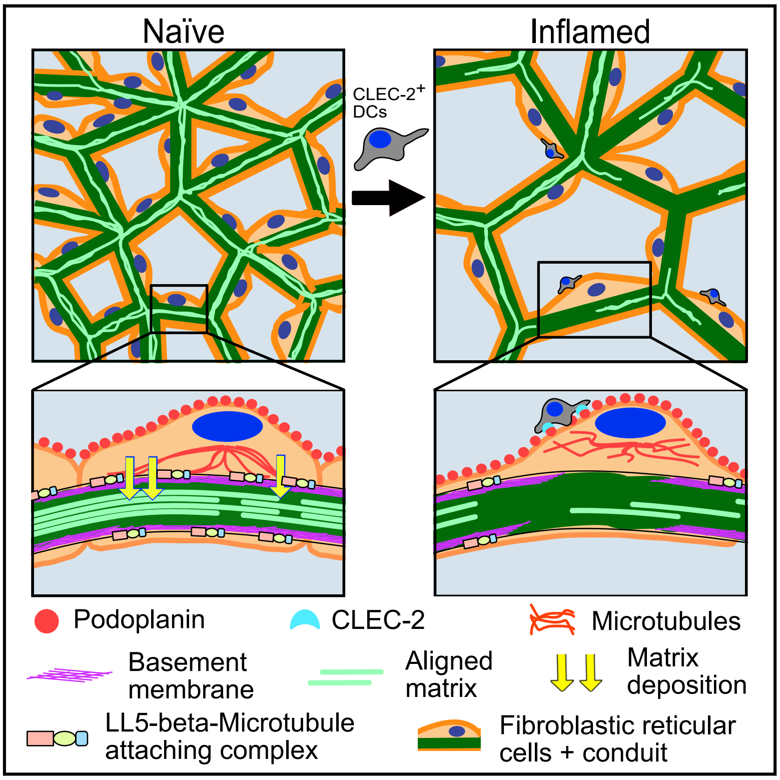

Authors

Victor G. Martinez, Valeriya Pankova, Lukas Krasny, ..., Paul H. Huang, Christopher J. Tape, Sophie E. Acton

Correspondence

s.acton@ucl.ac.uk

In Brief

Fibroblastic reticular cells control matrix production for lymph node conduit function. Martinez et al. show that matrix production is reduced and conduit flow is altered during lymph node expansion. Matrix deposition by fibroblastic reticular cells is controlled by CLEC-2/podoplanin signaling and directed unilaterally into conduit structures by LL5-β-tethered microtubules.

Highlights

- Conduit flow becomes locally intermittent during lymph node expansion
- Fibroblastic reticular cells use polarized microtubules to guide matrix deposition
- The CLEC-2/PDPN signaling axis controls conduit matrix composition
- Fibroblastic reticular cells reduce matrix production during lymph node expansion

Martinez et al., 2019, Cell Reports *29*, 2810–2822
November 26, 2019 © 2019 The Authors.
https://doi.org/10.1016/j.celrep.2019.10.103

OPEN ACCESS
CellPress

Cell Reports
Article

Fibroblastic Reticular Cells Control Conduit Matrix Deposition during Lymph Node Expansion

Victor G. Martinez,[1] Valeriya Pankova,[1] Lukas Krasny,[2] Tanya Singh,[3] Spyridon Makris,[1] Ian J. White,[4] Agnesska C. Benjamin,[1] Simone Dertschnig,[5] Harry L. Horsnell,[1] Janos Kriston-Vizi,[3] Jemima J. Burden,[4] Paul H. Huang,[2] Christopher J. Tape,[6] and Sophie E. Acton[1,7,*]
[1]Stromal Immunology Group, MRC Laboratory for Molecular Cell Biology, University College London, Gower Street, London WC1E 6BT, UK
[2]Division of Molecular Pathology, Institute of Cancer Research, 237 Fulham Road, London SW3 6JB, UK
[3]Bioinformatics Image Core, MRC Laboratory for Molecular Cell Biology, University College London, London WC1E 6BT, UK
[4]Electron Microscopy Facility, MRC Laboratory for Molecular Cell Biology, University College London, London WC1E 6BT, UK
[5]UCL Institute of Immunity and Transplantation, University College London, London NW3 2PF, UK
[6]Cell Communication Lab, Department of Oncology, University College London Cancer Institute, 72 Huntley Street, London WC1E 6DD, UK
[7]Lead Contact
*Correspondence: s.acton@ucl.ac.uk
https://doi.org/10.1016/j.celrep.2019.10.103

SUMMARY

Lymph nodes (LNs) act as filters, constantly sampling peripheral cues. This is facilitated by the conduit network, a tubular structure of aligned extracellular matrix (ECM) fibrils ensheathed by fibroblastic reticular cells (FRCs). LNs undergo rapid 3- to 5-fold expansion during adaptive immune responses, but these ECM-rich structures are not permanently damaged. Whether conduit flow or filtering function is affected during LN expansion is unknown. Here, we show that conduits are partially disrupted during acute LN expansion, but FRC-FRC contacts remain connected. We reveal that polarized FRCs deposit ECM basolaterally using LL5-β and that ECM production is regulated at transcriptional and secretory levels by the C-type lectin CLEC-2, expressed by dendritic cells. Inflamed LNs maintain conduit size exclusion, and flow is disrupted but persists, indicating the robustness of this structure despite rapid tissue expansion. We show how dynamic communication between peripheral tissues and LNs provides a mechanism to prevent inflammation-induced fibrosis in lymphoid tissue.

INTRODUCTION

Lymph node (LN) functional organization is formed and maintained by stromal cells (Link et al., 2007). Once thought to provide only the necessary scaffolds to support the architecture of the tissue, LN stromal cells are now recognized as key players in immunity (Buechler and Turley, 2018). Four main populations of stromal cells can be defined in LNs: podoplanin (PDPN)$^{-}$CD31^{+} blood endothelial cells (BECs), PDPN^{+}CD31^{+} lymphatic endothelial cells (LECs), PDPN^{+}CD31^{-} fibroblastic reticular cells (FRCs), and PDPN^{-}CD31^{-} double-negative cells (DNs) (Novkovic et al., 2018). Among these, the FRC population is the most abundant subset. A recent small conditional RNA sequencing (scRNA-seq) analysis showed that FRCs may contain different subpopulations with specific locations and functions (Rodda et al., 2018). FRCs form a connected 3-dimensional (3D) network that spans the T cell area and interfollicular regions of LNs. FRCs regulate the lymphocyte homeostasis (Cremasco et al., 2014; Link et al., 2007) and induction of peripheral tolerance (Dubrot et al., 2014; Fletcher et al., 2010; Lee et al., 2007). Furthermore, contraction through the FRC network also regulates LN size during immune responses. Interactions between FRC and C-type lectin-like receptor 2-expressing (CLEC-2^{+}) migratory dendritic cells (DCs) transiently inhibit PDPN-dependent actomyosin contractility during the acute phase of the immune response (Acton et al., 2014; Astarita et al., 2015), allowing rapid LN expansion.

LNs also function as filters for lymph-born antigens (Radtke et al., 2015; Randolph et al., 2017). Soluble antigens reach the LN first in a wave of draining-type diffusion ahead of a secondary wave of migratory antigen-presenting cells. Collected by lymphatic capillaries in the peripheral tissue, the lymph converges in afferent lymphatic vessels that merge with the LN capsule and flows within the subcapsular sinus (SCS). The lymph percolates through trabecular and cortical sinuses that flow into the LN medulla before leaving via efferent lymphatic vessels. A sample of low-molecular-weight molecules (<70 kDa) (Gretz et al., 2000; Nolte et al., 2003; Roozendaal et al., 2009; Sixt et al., 2005) is permitted to flow directly through the LN parenchyma within an intricate tubular system called the conduit network (Roozendaal et al., 2009). The conduit network is composed of bundled and aligned extracellular matrix (ECM) components enwrapped by FRCs, the main producers of ECM in the LN (Malhotra et al., 2012; Sixt et al., 2005; Sobocinski et al., 2010). No other fibrillar ECM structures are found in the LN parenchyma. While we are starting to understand how the FRC network reacts during LN expansion, it is not yet known how the non-cellular ECM components are remodeled and how rapid expansion of the LN may affect the function of the conduit. In other contexts, inflammation goes hand in hand with tissue remodeling. Injury-induced loss of the ECM is rapidly replenished by biogenesis and crosslinking (Xue and Jackson, 2015). Chronic inflammation, as it occurs in cancers or some viral

This is an open access article under the CC BY license (http://creativecommons.org/licenses/by/4.0/).

infections, induces deregulation of this process, leading to fibrosis (Wynn and Ramalingam, 2012). LN fibrosis can occur in tumor-draining LNs or in some cases of chronic viral infection (Riedel et al., 2016; Rohner et al., 2015; Schacker et al., 2006; Zeng et al., 2011); however, more commonly, LNs undergo a virtually unlimited number of inflammatory episodes throughout an individual's lifetime and LN fibrosis does not occur. Therefore, we hypothesized that a specific mechanism must be in place to both confine ECM secretion exclusively to the conduit and to avoid the accumulation of aberrant ECM during inflammation.

In this study, we focus on ECM remodeling by FRCs during LN expansion and the interconnection between the cellular and ECM components of the conduit network. We demonstrate depletion and disruption of ECM components of the conduit during acute LN expansion. We show that CLEC-2 binding to $PDPN^+$ FRCs modulates ECM production at both mRNA and protein levels. Furthermore, the CLEC-2/PDPN axis regulates polarized microtubule organization in FRCs to direct and contain ECM deposition.

RESULTS

Extracellular Matrix Components of the Conduit Are Reduced during Acute LN Expansion

To ask how ECM structures were maintained and remodeled during acute LN expansion, a period of rapid tissue growth, we first examined LN ECM structures in the steady state. Using the passive clarity technique (PACT) (Yang et al., 2014), we imaged collagen IV in intact naive inguinal LNs (Figure 1A; Video S1). This abundant basement membrane protein surrounded the LN vasculature and formed an intricate 3D connected network spanning the whole LN parenchyma (Malhotra et al., 2012; Sixt et al., 2005; Sobocinski et al., 2010), corresponding to the conduit network. Electron microscopy revealed the detail of condensed fibrillar bundles consisting of >200 collated fibers of ECM enwrapped by FRCs (Figure 1B). Co-staining of the basement membrane protein laminin and the FRC marker PDPN confirmed that in LN parenchyma, ECM structures are found exclusively associated with the FRC network forming the conduit (Bajénoff and Germain, 2009) and vasculature (Figure 1C).

We immunized mice with ovalbumin emulsified with incomplete Freund's adjuvant (IFA/OVA) and compared the density of collagen IV structures in LNs after 4 and 14 days (Figure 1D). We observed that the FRC network appeared stretched but connected at day 4 and that collagen IV structures were less prominent. Using podoplanin staining as a mask, we quantified collagen IV intensity exclusively within the FRC cellular network and found a progressive loss and disruption of conduit matrix over time in inflamed LNs (Figure 1E). We obtained similar results for collagen I and collagen VI (Figures 1F and 1G), indicating that while the FRC cellular network remained connected and intact, the accompanying ECM components of the conduit remained associated with the FRCs but were no longer replete.

CLEC-2 Binding Regulates ECM Production by FRCs

FRCs rapidly change their morphology and network architecture in response to $CLEC\text{-}2^+$ migratory DCs (Acton et al., 2014). We hypothesized that the remodeling of the cellular network may also affect the remodeling of the associated ECM downstream of the same DC/stromal contacts. We stimulated FRCs *in vitro* with CLEC-2-Fc recombinant protein and compared transcriptional profiles by RNA-seq (Figures 2 and S1). Bulk analysis of the transcriptomic data comparing 6- and 24-h CLEC-2-Fc treatment revealed that CLEC-2-Fc induced a transient and largely reversible gene regulation response in FRCs (Figure S1A). This transient transcriptional regulation follows kinetics similar to how CLEC-2 inhibits PDPN-dependent contractility in FRCs (Acton et al., 2014). Gene Ontology analysis (Mi et al., 2013, 2017) showed that genes encoding proteins in the extracellular space/region were most enriched among CLEC-2-Fc-regulated genes (Figure 2A). Using the Matrisome database (Naba et al., 2012, 2016, 2017) of all ECM proteins and associated factors, we found that FRCs expressed 570 of 743 matrisome genes *in vitro*, of which 75 (13%, across all matrisome categories) were regulated >2-fold 6 h after CLEC-2-Fc binding (Figures 2B and S1B).

FRCs regulated 35 core matrisome genes (>2-fold) in response to CLEC-2-Fc, including 1 collagen (*Col8a2*), 23 glycoproteins, and 6 proteoglycans (Figure 2C). The downregulated glycoproteins were mostly associated with cell-matrix adhesion and migration, including *Nov*, *Sparcl1*, *Ntn1*, *Igfbp5*, *Ndnf*, *Spon2*, *Efemp1*, and *Fbln7* (de Vega et al., 2016; Ellis et al., 2003; Gagliardi et al., 2017; Jia et al., 2005; Ohashi et al., 2014; Song et al., 2011; Sureshbabu et al., 2012; Yin et al., 2018). The glycoprotein genes induced had more pleiotropic roles, such as growth factor signaling (*Ctgf*, *Tsku*, *Wisp1*, and *Ltbp2*) (Enomoto et al., 2018; Lee et al., 2010; Niimori et al., 2014; Ono et al., 2018) or immunomodulation (*Spp1*, *Tnc*, and *Crispld2*) (Castello et al., 2017; Murdamoothoo et al., 2018; Wang et al., 2009). The regulation of proteoglycan expression by CLEC-2-Fc was more evident at 24 h, suggesting that CLEC-2-Fc may be indirectly regulated (e.g., CTGF/CCN2 represses *Ogn*, *Dcn*, *and Omd*; Seher et al., 2011). CLEC-2-Fc increased the expression of *Prg4*, which inhibits synoviocyte cell/matrix adhesion (Qadri et al., 2018).

Most of the 17 ECM-affiliated genes that were regulated by CLEC-2-Fc were linked to cytoskeleton regulation (Afratis et al., 2017; Casazza et al., 2007; Hamm et al., 2016) (Figure 2C), including members of the semaphorin-plexin system, which provides guidance cues for migration (Casazza et al., 2007). Known to inhibit axonal growth (Casazza et al., 2007), the expression of *Sema6c*, *Sema5a*, *Sema3f*, *Sema3e*, *Sema3a*, and *Sema3d* were reduced upon CLEC-2-Fc treatment, hinting that FRCs may spread using similar mechanisms. Of note, CLEC-2-Fc induced the expression of *Sema7a*, which represses ECM production in other fibroblasts (Esnault et al., 2017).

CLEC-2-Fc regulation of 23 ECM regulators (Figure 2C) mainly affected protease inhibitors, including the upregulation of *Serpine1*, *Timp1*, and *Timp3,* key in the negative regulation of matrix metalloproteinase (MMP) activity (Flevaris and Vaughan, 2017; Zhai et al., 2018). Also upregulated are *Sulf1* and *Tll1*, which are involved in ECM biogenesis (Kessler et al., 1996; Nagamine et al., 2012). CLEC-2-Fc repressed the expression of several ECM regulator genes with prominent roles in ECM degradation: *Hyal1* (hyaluronidase-1) (Harada and Takahashi, 2007), *Agt* (SERPINA8/angiotensinogen) (Rodrigues-Ferreira et al., 2012), *Htra3* (Bost et al., 1998; Tocharus et al., 2004), *Adamts1*

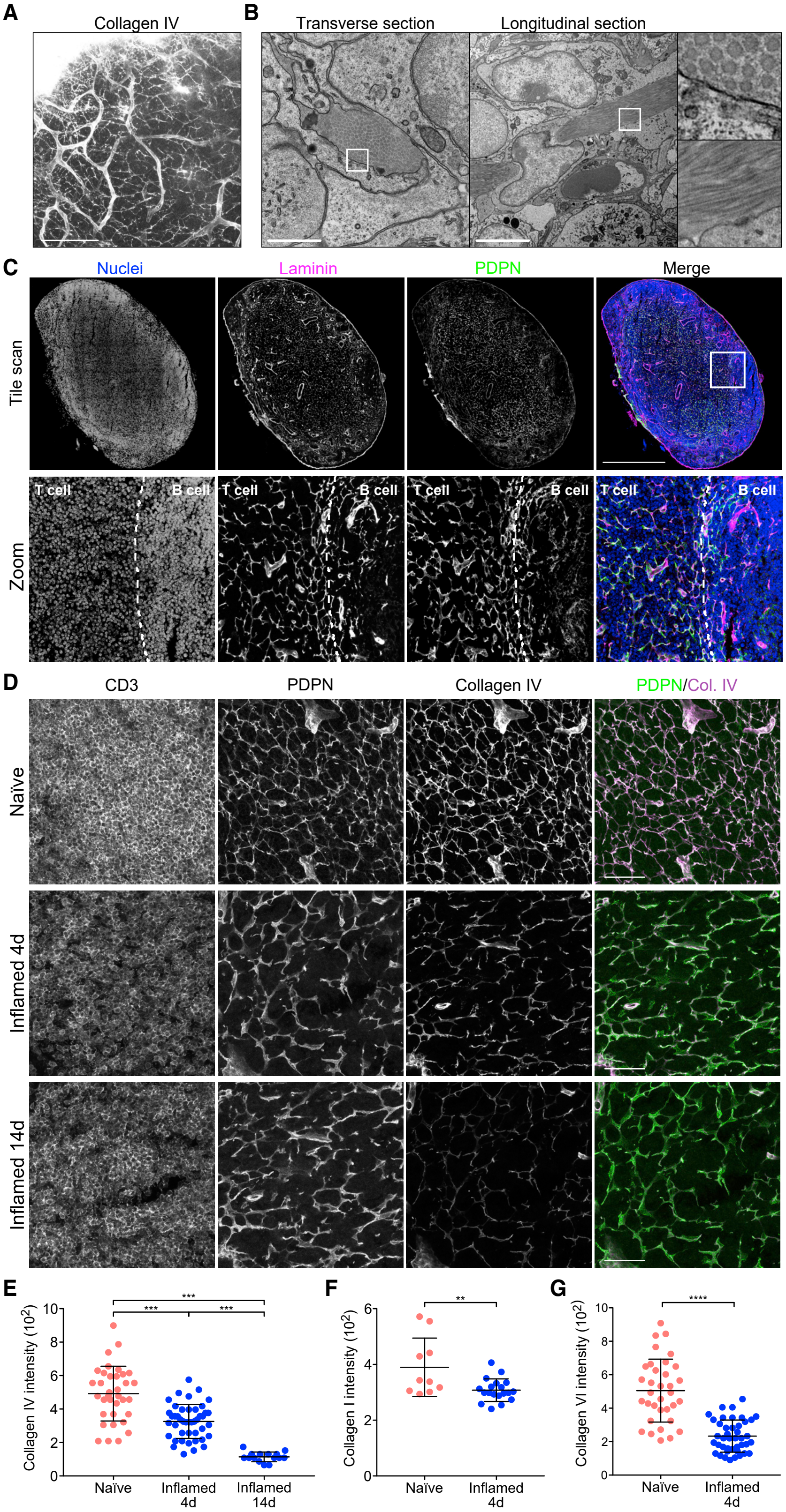

Figure 1. Conduit Composition Changes during LN Expansion

(A) Representative maximum z stack projection of intact naive lymph node stained for collagen IV using PACT. Scale bar, 100 μm.

(B) Representative transmission electron microscopy of naive LNs. Scale bars, 5 μm.

(C) Representative cryosection of a naive LN. Tile scan (top) and magnification (bottom). Dashed line indicates T and B cell boundaries. Scale bars, 500 μm (tile scan).

(D) Cryosections of the T cell area naive and inflamed LNs immunized with IFA/OVA. Scale bars, 100 μm.

(E) Quantification of collagen IV in the PDPN$^+$ network within the T cell area. Each point represents the median gray intensity of region of interest, from 5 biological replicates. Error bars represent means and SDs. ***$p < 0.0005$.

(F) Quantification of collagen I within the PDPN$^+$ network. Each point represents the median gray intensity of region of interest, from 3 biological replicates. Error bars represent means and SDs. **$p < 0.005$.

(G) Quantification of collagen VI within the PDPN$^+$ network. Each point represents the median gray intensity of region of interest, from 5 biological replicates. Error bars represent means and SDs. ****$p < 0.0001$.

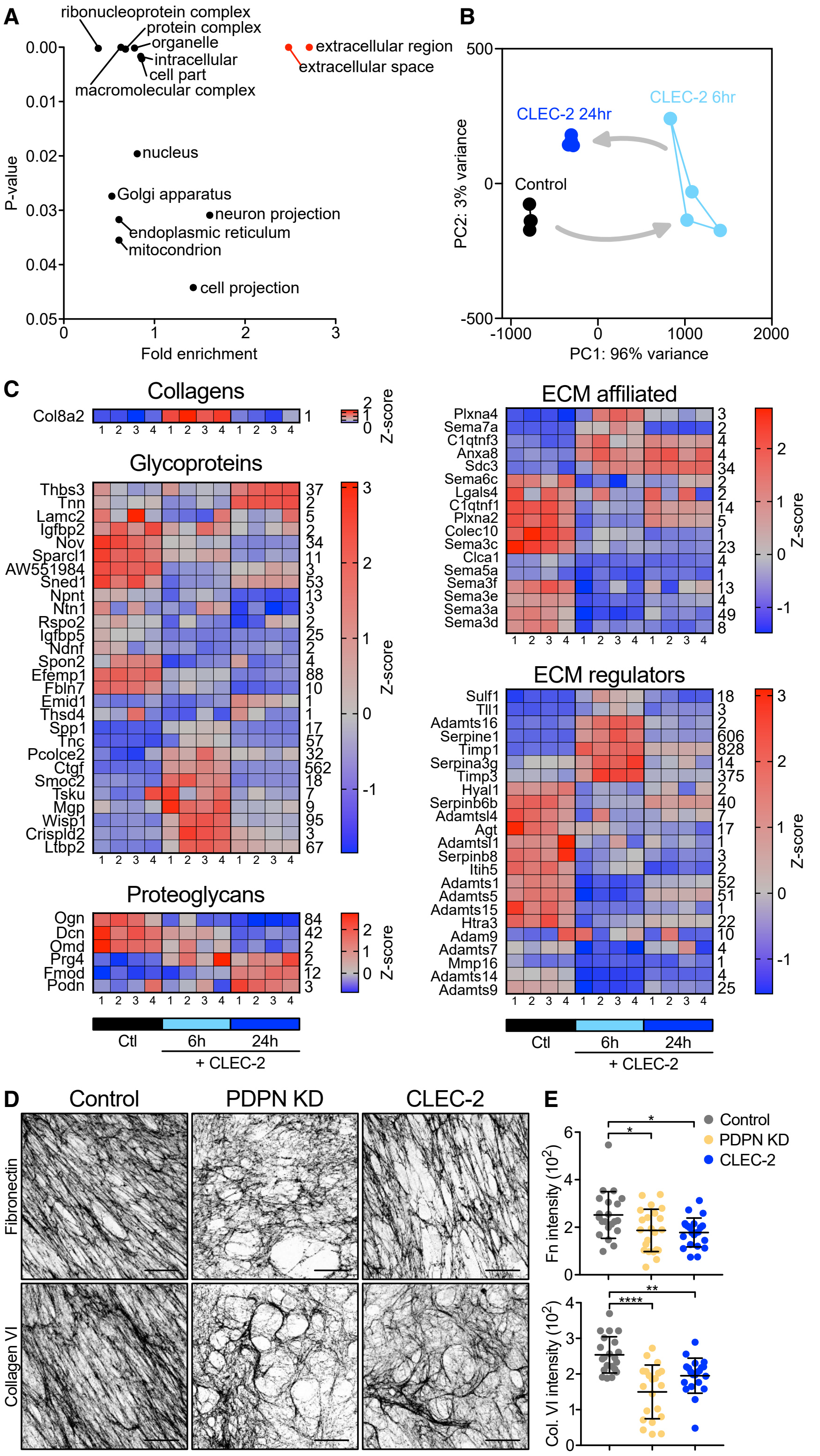

Figure 2. Effects of CLEC-2 on ECM Production by FRCs

(A) Gene Ontology analysis, genes regulated by CLEC-2-Fc ≥2-fold. RNA-seq data from 4 biological replicates per condition.

(B) CLEC-2-Fc-regulated matrisomal gene cluster in a principal-component analysis (PCA) space. Arrows indicate time course.

(C) Heatmaps of matrisomal genes regulated ≥2-fold by CLEC-2-Fc. Four biological replicates for each condition are shown. Color-coding represents *Z* score; row average is indicated (right).

(D) Fibronectin (top) and collagen VI (bottom) representative immunofluorescence staining of *in vitro* FRC-derived matrices. Maximum z stack projections; scale bars, 20 μm.

(E) Median gray intensity for ECM components. Each dot represents a region of interest, combined from 3 biological replicates. Error bars represent means and SDs. *p < 0.05, **p < 0.005, ***p < 0.0005, one-way ANOVA, Tukey's multiple comparisons test.

(Porter et al., 2005), *Adamts5* (Evanko et al., 2012), *Adamts7* (Riessen et al., 2001), *Adamts9* (Yoshina et al., 2012), *Adamts15* (Dancevic et al., 2013), *Adam9* (Roychaudhuri et al., 2014), and *Mmp16* (Roth et al., 2017).

These data indicate that FRCs can substantially alter their transcriptional profile following CLEC-2 binding and that transcriptional regulation may play an important role in ECM remodeling and cell matrix adhesion in FRCs. Furthermore, the induction of protease inhibitors plus the repression of proteases suggest that the observed loss of ECM within the conduit during LN expansion (Figure 1D) is unlikely to be due to degradation by FRCs. Furthermore, since we observed that collagens (I, IV, and VI) are reduced *in vivo* in inflamed LNs (Figure 1D) but were not transcriptionally regulated by CLEC-2, this transcriptional regulation alone cannot fully explain the reduced ECM observed (Figure 1D).

To investigate whether the CLEC-2/PDPN signaling axis regulates ECM production at the protein level, we undertook a proteomic analysis of FRC-derived matrices *in vitro* (Figure S2). We generated CLEC-2-Fc-secreting FRCs to allow constant CLEC-2 stimulation and compared them to PDPN-depleted FRCs (PDPN knockdown [KD]) (Acton et al., 2014) and a control FRC cell line. Mass spectrometry analysis detected a similar number of proteins in all 3 FRC cell lines, in which 96 proteins were matrisomal proteins, with almost 90% overlap among the samples (Figure S2A). PDPN depletion phenocopies the loss of contractility induced by CLEC-2 binding (Acton et al., 2014); in contrast, when comparing ECM protein production, PDPN KD FRCs appeared qualitatively different from either

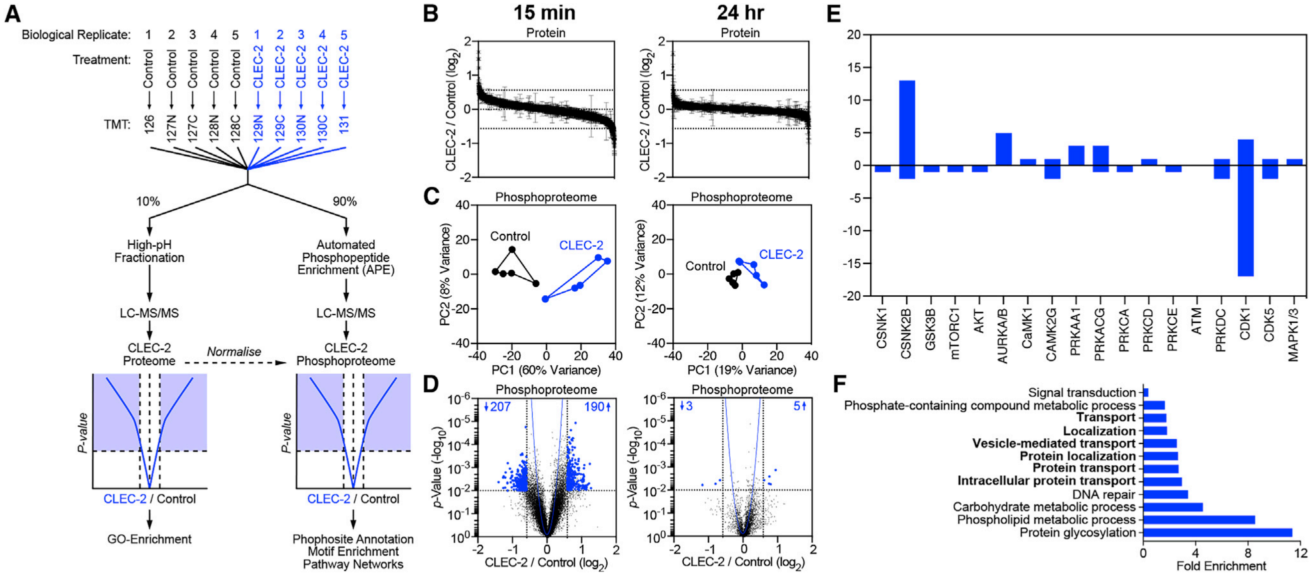

Figure 3. Phosphoproteomics of CLEC-2-Fc-Treated FRCs

(A) Experimental setup, comparison of 5 control (untreated) and 5 CLEC-2-Fc-treated FRC cell lysates (biological replicates).

(B) Waterfall plots showing proteome regulation by CLEC-2-Fc.

(C) Control and CLEC-2-Fc-treated phosphoproteomes cluster in a PCA space.

(D) Volcano plots showing statistical regulation of the CLEC-2-Fc-treated FRC's phosphoproteome (n = 5, two-tailed t test, Gaussian regression). The number of regulated phosphosites is indicated.

(E) Empirical parent kinase analysis. The bars represent the number of targets for kinases. Positive and negative values indicate higher or lower phosphorylation in CLEC-2-Fc-treated cells.

(F) Gene Ontology analysis for biological processes. Each bar represents a biological process significantly enriched by binomial analysis.

control or CLEC-2-Fc-secreting FRCs (Figure S2B). PDPN KD FRC-derived matrices showed an overall reduction in ECM components, whereas CLEC-2-Fc-secreting FRCs and controls were more closely aligned (Figure S2C). This suggests that the loss of PDPN expression is not equivalent to CLEC-2 modulation of PDPN function in the case of matrix production.

While the CLEC-2/PDPN signaling axis influenced both matrix transcription (Figures 2A–2C) and protein production (Figure S2), how these changes translated to fibril formation, relevant to conduit remodeling *in vivo*, was still unclear. Staining of decellularized FRC-derived matrices for fibronectin and collagen VI showed that ECM structures formed by CLEC-2-Fc-secreting FRCs appeared disorganized compared to controls, with lower alignment, large empty spaces, and lower median intensity of matrix fibers (Figures 2D and 2E). In this functional assay, PDPN KD FRCs phenocopied the effect of CLEC-2-Fc in matrix deposition and organization. These experiments demonstrate that CLEC-2/PDPN signaling regulates ECM remodeling at multiple levels, gene expression, protein production, and secretion and fibril arrangement. These results also indicate that PDPN expression by FRCs is a key requirement for FRCs to produce, deposit, and align ECM components, and that this process is modulated by CLEC-2.

Signaling Cascades Regulated by CLEC-2 in FRCs

The above results suggest that additional cellular mechanisms are likely to regulate ECM deposition in FRCs. To address the CLEC-2/PDPN-dependent signaling cascades controlling ECM organization, we performed an unbiased phosphoproteomics analysis of FRCs by tandem mass tag mass spectrometry (TMT-MS) (Tape et al., 2016) (Figure 3A). Control FRCs were stimulated with CLEC-2-Fc for 15 min or 24 h to capture immediate and late signaling responses. As expected, the total protein levels did not differ significantly following treatment (Figure 3B). However, phosphoproteome analysis revealed that CLEC-2-Fc induced a rapid and transient signaling response in FRCs (Figure 3C). At 15 min, ≈400 phosphorylation sites were regulated by CLEC-2-Fc (Figure 3D), corresponding to 77 proteins. In contrast, after 24 h, only 8 phosphorylation sites corresponding to 6 proteins were regulated compared to controls, confirming the transient and reversible nature of responses to CLEC-2/PDPN engagement (Figure 3D).

To elucidate signaling cascades regulated by CLEC-2, we performed kinase target analysis (15 min dataset). We found that CSNK2B and CDK1 regulated the highest number of predicted targets (Figure 3E). Gene Ontology analysis of hits (Mi et al., 2013, 2017) highlighted intracellular protein transport pathways (Figure 3F) that were relevant to the transport and deposition of cargo such as ECM components. However, using a GFP-based assay, we found no reduction in protein secretion in either CLEC-2-Fc-treated or PDPN KD FRCs (Figure S3). Nevertheless, the impaired ECM deposition that was observed in both CLEC-2-Fc-treated and PDPN KD FRCs (Figure 2D) prompted a closer look at the phosphoproteomic data. Secretion of large cargo proteins such as ECM components requires vesicle transport via such cytoskeletal structures as the microtubule network

(Noordstra and Akhmanova, 2017). We found that several key regulators of microtubule function were post-translationally modified by CLEC-2-Fc stimulation, including cytoplasmic linker protein 170 (CLIP-170), cytoplasmic dynein heavy chain 1, and pleckstrin homology-like domain family B member 2 (LL5β). While the direct function of these regulatory sites has not been previously described, these data presented strong evidence that CLEC-2 altered the organization of microtubules in FRCs, a possible regulatory mechanism of ECM deposition in LNs.

CLEC-2 Binding Controls Microtubule Organization in FRCs via LL5β

LL5β forms complexes that attach plus ends of microtubules to the cell membrane, providing a secretory pathway for localized exocytosis, which facilitates apicobasal cell polarity in epithelial cells (Hotta et al., 2010; Lansbergen et al., 2006; Noordstra and Akhmanova, 2017; Stehbens et al., 2014). Given that ECM components in LNs are tightly compartmentalized by FRCs within the conduit, we reasoned that FRCs may be using a similar pathway to secrete ECM. We therefore examined the role of LL5β in ECM deposition by FRCs. We found that both CLEC-2-Fc treatment and PDPN KD reduced LL5β protein and mRNA levels in FRCs (Figures 4A and 4B). We attempted unsuccessfully to overexpress a phosphomimetic mutant (LL5β S465E) in FRCs, leading us to hypothesize that phosphorylation of LL5β at S465 may target LL5β for degradation.

Control FRCs clustered LL5β at the cell periphery (Figure 4C); however, this accumulation was absent in PDPN KD FRCs and more cytoplasmic in CLEC-2-Fc-treated cells (Figures 4C and 4D). The reduced peripheral localization of LL5β coincided with the lower density of microtubules at the cell periphery in PDPN KD and CLEC-2-Fc-treated cells (Figure 4E). We confirmed the colocalization of LL5β with cortical microtubules in control FRCs, in which LL5β$^+$ areas presented a higher microtubule density compared to areas lacking LL5β (Figure 4F). To investigate whether LL5β was required for microtubule attachment to the cortex in FRCs, we silenced LL5β expression using small interfering RNA (siRNA) (Figure 4G), which resulted in a corresponding loss of microtubules from the periphery (Figure 4H). LL5β-silenced FRCs also showed significantly reduced matrix deposition (Figures 4I and 4J), phenocopying the disrupted matrix generated following either CLEC-2-Fc treatment or PDPN depletion (Figure 2H) and confirming that LL5β is necessary for ECM deposition in FRCs.

Loss of FRC Adhesion and Reorganization of Microtubule Networks

LL5β is recruited to mature focal adhesion complexes, which require Rho-kinase (ROCK)/myosin-mediated contractility (Katoh et al., 2001; Stehbens et al., 2014). Since the CLEC-2/PDPN signaling axis inhibits actomyosin contractility in FRCs (Acton et al., 2014), we predicted it may also alter FRC adhesion to the underlying conduit and therefore inhibit the localization of LL5β and microtubules to the cell cortex. We compared the structure and localization of focal adhesions (p-paxillin) and LL5β between FRC cell lines. In controls, LL5β clustered directly adjacent to elongated mature focal adhesions (Stehbens et al., 2014) (Figures 5A and 5B). However, CLEC-2-Fc-treated and PDPN KD cells presented significantly shorter focal adhesions (Figures 5A and 5B). This was phenocopied by the direct inhibition of ROCK (Y-27632) (Figures 5A and 5B). This result shows that when focal adhesion maturation is disrupted, there is a concordant loss of LL5β clustering, linking actomyosin contractility, cell matrix adhesion, and LL5β recruitment in an integrated mechanism (Figures 5A and 5B). To test these linked outcomes in a more physiological assay, we stimulated FRCs with either control or CD11c$^{\Delta CLEC\text{-}2}$ bone marrow-derived dendritic cells (BMDCs). Cultured alone, FRCs displayed prominent F-actin stress fibers and mature elongated focal adhesions to which microtubule bundles docked in abundance (Figures 5C and 5D). Interaction with control (CLEC-2$^+$) BMDCs induced the loss of actin stress fibers, shorter focal adhesions, and lower microtubule density at the periphery (Figures 5C and 5D). This change in FRC morphology and function was not observed with CD11c$^{\Delta CLEC\text{-}2}$ BMDCs (Figures 5C and 5D), demonstrating that DC-induced inhibition of actomyosin contractility and microtubule localization requires CLEC-2.

We next asked whether LL5β directs the microtubule-mediated deposition of matrix components in the FRC network *in vivo*. High-resolution imaging of LN tissue revealed that in naive LNs, the entire FRC network expressed high levels of LL5β, and its localization was always polarized inward toward the ensheathed conduit (Figure 5E). In contrast, in inflamed LNs, we observed many regions of the FRC network that lacked polarized localization of LL5β, coinciding with the loss of laminin in the same region (Figure 5E). This is a direct translation of the *in vitro* studies that predicted the loss of LL5β when matrix adhesion is lost (Figures 4 and 5). These data require us to consider FRCs as polarized cells, exhibiting apical and basolateral cell polarity similar to epithelial sheets (Noordstra and Akhmanova, 2017), but enwrapping the conduit similar to Schwann cells enwrapping nerve fibers (Tricaud, 2018). The inner surface of the FRC adheres to the conduit and recruits LL5β for ECM secretion, while the outer surface of the FRC excludes ECM, allowing optimal interaction with lymphocytes and antigen-presenting cells. Polarized and localized exocytosis in FRCs, directed by LL5β, can mechanistically explain how ECM components are exclusively found within the conduit and not elsewhere in the LN parenchyma.

Conduit Size Exclusion and Flow Persist during LN Expansion

We next asked how remodeling the ECM would affect conduit function. We compared the flow of fluorescently labeled dextrans through the LN sinuses and conduits of naive and acutely inflamed LNs (5 days post-immunization). It has been previously shown that 10-kDa dextrans can flow through the conduit, while those >70 kDa are too large and are retained in the subcapsular sinus (Gretz et al., 2000; Nolte et al., 2003; Roozendaal et al., 2009; Sixt et al., 2005). In accordance with previous studies, the 500 kDa dextran was excluded from all but the most proximal branches of the conduit in both naive and inflamed LNs (Figure S4A), meaning that the filtering function and size exclusion of the conduit network are maintained during LN expansion (Figure S4A). Furthermore, these results indicated that ECM loss within the network did not impede the overall flow of small soluble molecules through LNs.

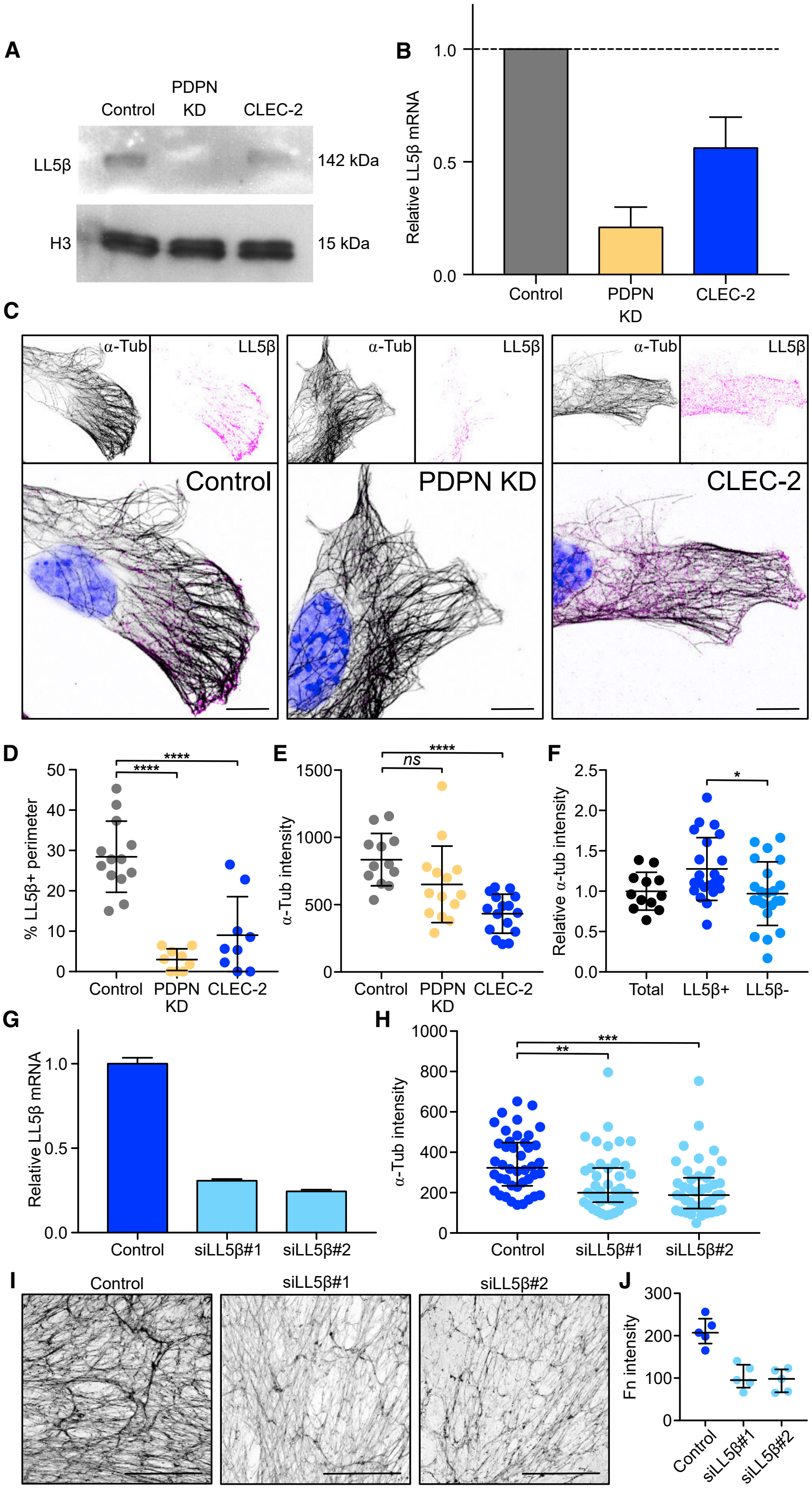

Figure 4. Regulation of Microtubule Organization by CLEC-2 via LL5β

(A) Representative western blots showing LL5β expression in FRC cell lines.

(B) Expression of LL5β mRNA relative to control FRCs by qPCR. Error bars represent means and SDs of 2 biological replicates.

(C) Immunofluorescence of FRC cell lines *in vitro*. Maximum z stack projections. Scale bars, 10 μm.

(D) Quantification of LL5β coverage in FRC cell lines as a percentage of the total perimeter. Each dot represents 1 cell. Error bars represent means and SDs. ****$p < 0.00005$, one-way ANOVA, Tukey's multiple comparisons test.

(E) α-Tubulin intensity in the cortical area (10 μm from the edge) of FRC cell lines. Each dot represents 1 cell. Error bars represent means and SDs. ****$p < 0.00005$, one-way ANOVA, Tukey's multiple comparisons test. NS, not significant.

(F) α-Tubulin intensity in the cortical area (10 μm from the edge) in $LL5\beta^{+}$ and negative areas in control FRCs relative to the total area. Black dots represent cells and blue dots represent cell areas. Error bars represent means and SDs. *$p < 0.05$, one-way ANOVA, Tukey's multiple comparisons test.

(G) Expression of LL5β mRNA by qPCR in LL5β siRNA transfected FRCs ($n = 2$). Error bars represent means and SDs.

(H) αTubulin intensity in the cortical area (10 μm from the edge). Each dot represents 1 cell. Error bars represent means and SDs. **$p < 0.005$, ***$p < 0.0005$, one-way ANOVA, Tukey's multiple comparisons test.

(I) Representative *in vitro* cell-derived matrices from LL5β KD FRCs, decellularized and stained for fibronectin. Maximum z stack projections. Scale bars, 100 μm.

(J) Median gray intensity for fibronectin staining. Each dot represents a different region of interest, from 2 biological replicates. Error bars represent means and SDs.

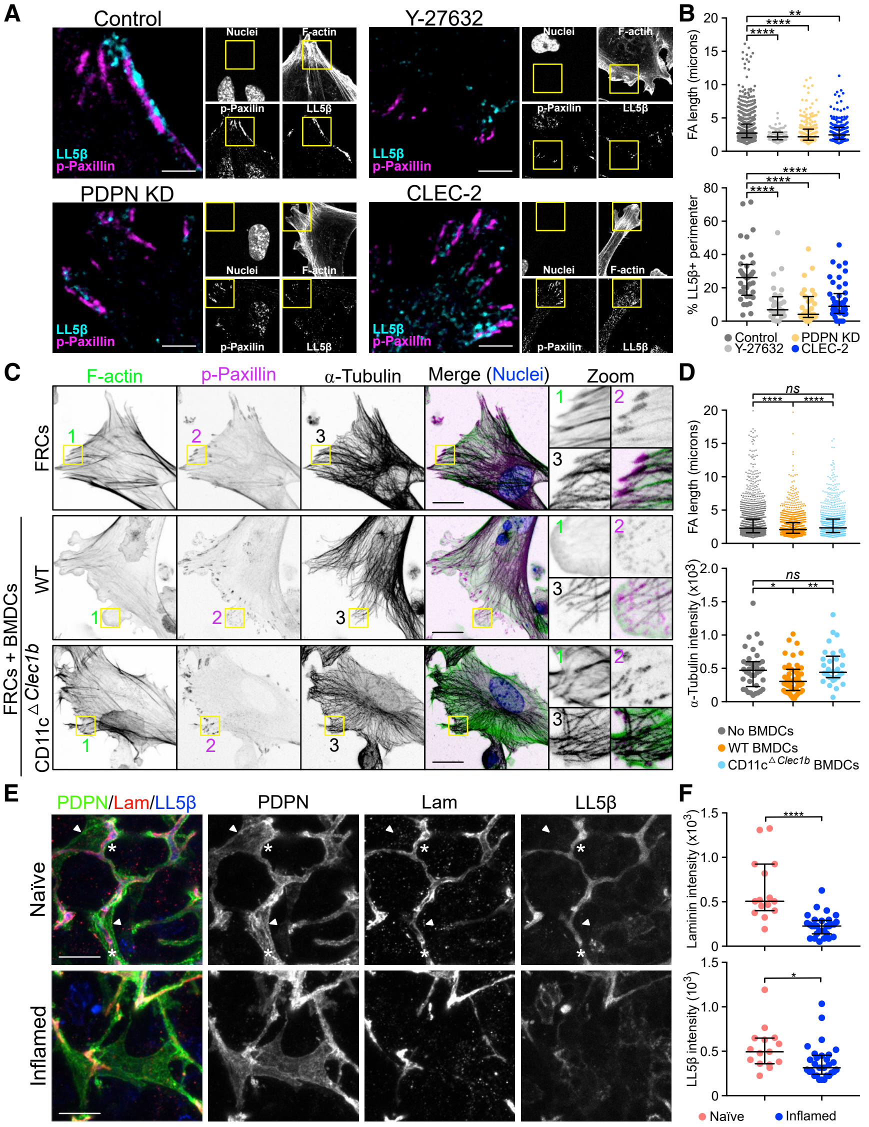

Figure 5. Focal Adhesion, Microtubule Organization, and Contractility in FRCs

(A and B) Representative immunofluorescence of FCR cell lines untreated and control FRCs treated with Y-27632 ROCK inhibitor. (A) Maximum z stack projections of representative images are shown. Scale bars, 5 μm. (B) Quantification of FA length from p-paxillin staining and LL5β coverage as a percentage of the total perimeter. Dots represent FAs (top) or single cells (bottom) from 2 biological replicates. $^{**}p < 0.005$, $^{****}p < 0.00005$, one-way ANOVA, Tukey's multiple comparisons test. Error bars represent SDs.

(C) Immunofluorescence of FRC/DC cocultures control or CD11c$^{\Delta CLEC-2}$ BMDCs. Maximum z stack projections. Scale bars, 20 μm.

(D) Quantification of FA length and α-tubulin intensity in the cortical area (10 μm from the edge). Dots represent FAs (top) or single cells (bottom) from 3 biological replicates. $^{*}p < 0.05$, $^{**}p < 0.005$, $^{****}p < 0.00005$, one-way ANOVA, Tukey's multiple comparisons test. NS, not significant. Error bars represent SDs.

(E) Representative immunofluorescence of cryosections of naive and inflamed LNs immunized with IFA/OVA. Maximum z stack projections. Scale bars, 20 μm. Asterisks and arrowheads indicate conduit-associated and conduit-independent surfaces, respectively, of FRCs.

(F) Quantification of the indicated conduit components within the PDPN network. Each dot represents the median gray intensity of a different region of interest from 5 biological replicates. Error bars represent means and SDs. $^{*}p < 0.05$, $^{****}p < 0.00005$, unpaired t test.

However, looking in more detail, we noticed the presence of numerous gaps or interruptions in dextran flow (10 kDa) through inflamed LNs, in which only <20% of the FRCs network was dextran^{+} in inflamed LNs (Figure 6A, right panel). We found that the presence of dextran flow in inflamed LNs perfectly correlated with FRCs that had maintained both polarized LL5β and laminin (Figure 6B), indicating that conduit function is dysfunctional in many sections of the FRC network. However, despite the substantial reduction in conduit structures during acute expansion, the overall global distribution of small molecules through the conduit is maintained, reinforcing the robustness of the FRC network (Novkovic et al., 2016).

Conduit Sampling by CD11b^{+} Cells Is Increased in Inflamed LNs

Soluble antigen flow through the conduit ensures controlled antigen sensing by LN resident cells (Gretz et al., 2000; Nolte et al., 2003; Palframan et al., 2001; Roozendaal et al., 2009; Sixt et al., 2005). We asked whether the interruptions in conduit flow observed in inflamed LNs would affect antigen uptake. Using flow cytometry, we found a similar percentage of dextran^{+} cells in inflamed LNs compared to naive (Figure 6C) within 90 min post-dextran injection. The total number of dextran^{+} cells was approximately doubled, but this is in line with the increase in LN cellularity (Figure 6Di). We found increased numbers of dextran^{+} cells within both MHC-II^{-} and MHC-II^{+} populations (Figure 6Dii), with MHC-II^{+} cells representing 80% of all dextran^{+} cells (Figure S5B), the majority of these being CD11c$^{-/low}$CD11b^{+} (monocytes/macrophages) cells in inflamed LNs (Figure 6Diii). Upon examination of the tissue sections, we found that the increased numbers of dextran^{+} monocytes/macrophages in inflamed LNs may result from the active recruitment of more CD11b^{+} monocytes/macrophages to conduits (Figure 6E). We observed double the number of CD11b^{+} cells interacting with the FRC network per area compared to naive controls (Figures 6E and 6F). Overall, we find that despite the local loss of conduit function (Figure 6A), the global robustness of the conduit network together with the increased monocytes/macrophage recruitment to conduits is able to maintain soluble antigen uptake throughout LN expansion.

OPEN ACCESS
CellPress

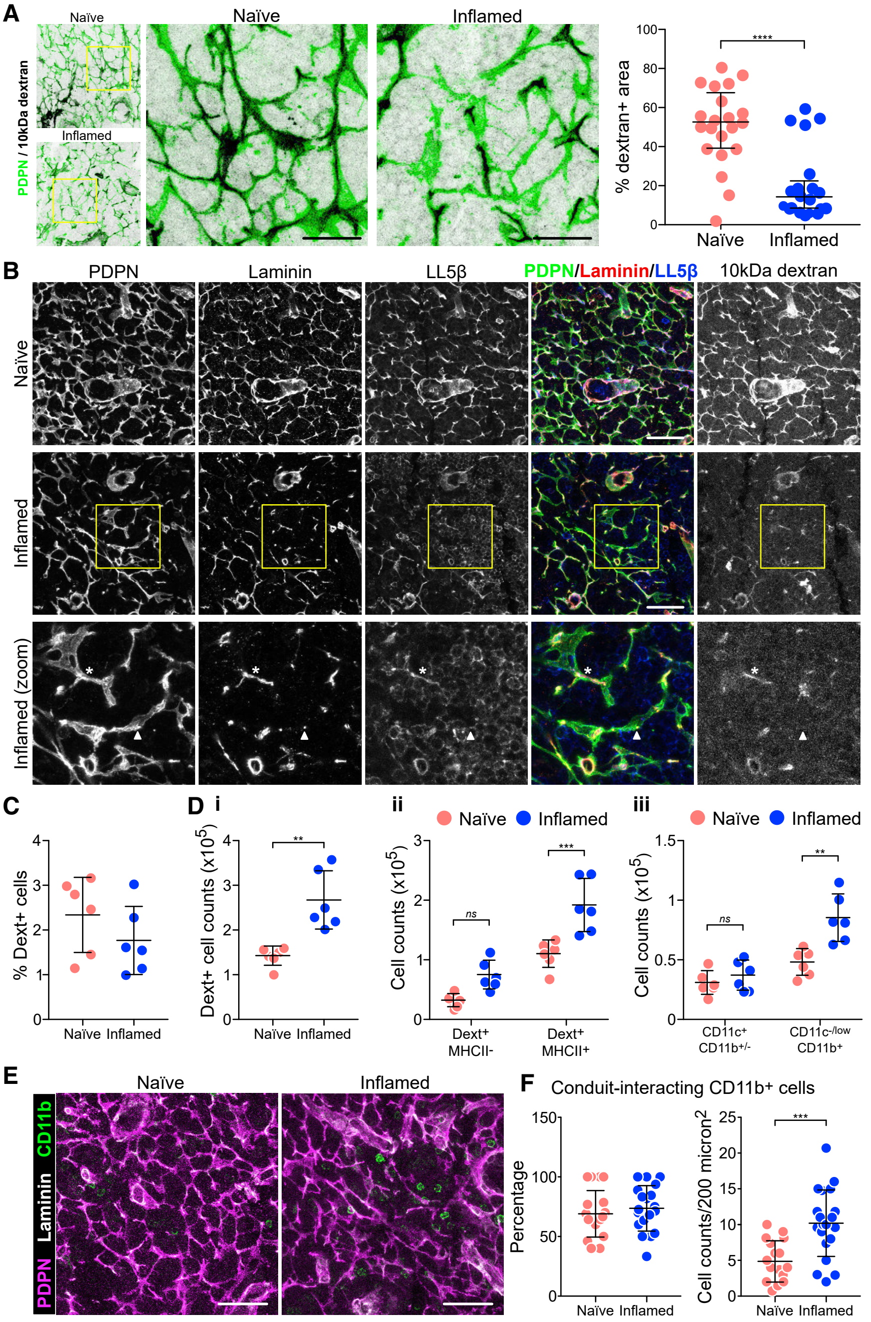

Figure 6. Conduit Flow and Antigen Uptake in Inflamed LNs

Mice were immunized by the subcutaneous injection of IFA/OVA on the right flank. Five days later, fluorescently labeled 10 kDa dextran was injected on both flanks.

(A) Immunofluorescence of 20-μm-thick cryosections of naive and inflamed draining LNs 30 min post-dextran injection. Maximum z stack projections. Scale bars, 40 μm. Graph shows the percentage of dextran$^+$ areas within the PDPN$^+$ network. Each dot represents a different region of interest. Regions of interest are collated from 6 individual mice per group. Error bars represent means and SDs. ****$p < 0.0001$, unpaired t test.

(B) Immunofluorescence of 20-μm-thick cryosections of naive and inflamed draining LNs 30 min post-dextran injection. Maximum z stack projections. Scale bars, 20 μm. The asterisk indicates a portion of the FRC network with all conduit components plus dextran flow. The arrowhead indicates a portion of the FRC network in which the conduit is not present and dextrans are not flowing.

(C) Percentage of dextran$^+$ cells as quantified by flow cytometry 90 min after dextran injection. Error bars represent means and SDs.

(D) The number of dextran$^+$ cells per LN (i); the number of MHCII$^+$ dextran$^+$ cells (ii); the number of dextran$^+$ cells within myeloid subsets (iii). Each dot represents an individual mouse (n = 6). Error bars represent means and SDs.

(E) Representative images of CD11b$^+$ myeloid cells in cryosections of the fibroblastic reticular network. Scale bars, 20 μm.

(F) Quantification of the percentage (left) and the number (right) of CD11b$^+$ myeloid cells interacting with conduit structures from regions of interest from 5 biological replicates. Error bars represent means and SDs.

DISCUSSION

Our results show that conduit flow within the T cell area conduit network is locally compromised during adaptive immune responses and that ECM components are lost or effectively diluted during early LN expansion. As a result, we observe discontinuous conduit flow within inflamed LNs, indicating areas of potential leakage. However, if conduits were leaking soluble antigens, then we may expect increased indiscriminate uptake by the many phagocytic cells within the tissue. Instead, we see that dextrans continue to be sampled from the conduits by a subset of myeloid cells and that an increased number of CD11b$^+$ monocytes/macrophages are recruited to interact with the FRC network. We conclude that the conduit network is sufficiently robust in offering alternative routes around the dysfunctional sections. Conduit size exclusion requires plasmalemma vesicle-associated protein (PLVAP) expression by the lymphatic endothelial cells lining the sinus (Rantakari et al., 2015). Our study provides further evidence that this barrier remains intact during early LN expansion, continuing to protect lymphoid tissue from intact pathogens.

Previous work has described the complex architecture of the conduit network in the steady state, and it is known that FRCs produce and organize ECM components (Malhotra et al., 2012; Roozendaal et al., 2009; Sixt et al., 2005; Sobocinski et al., 2010). We show that FRCs exhibit a type of apical/basolateral cell polarity and organize microtubule networks to direct ECM

deposition unilaterally into conduit structures. Recruitment of LL5β facilitates the docking and attachment of the plus ends of microtubules to the cell membrane at sites of FRC-matrix adhesion and enables matrix deposition.

We found that CLEC-2 binding to FRCs regulates their matrix remodeling at both transcriptional and protein levels and also regulates FRC adhesion, contractility, and microtuble networks via LL5β recruitment, transiently inhibiting matrix deposition. This mechanism would explain how LNs are able to avoid the aberrant accumulation of excess ECM while enduring repeated episodes of inflammation. We have previously shown that CLEC-2$^+$ DCs inhibit PDPN-dependent contractility in FRCs (Acton et al., 2014). This weakens FRC adhesion and in turn inhibits the recruitment of LL5β to the conduit. LL5β expression is essential for FRCs to organize microtubules and form ECM matrices, and it is exclusively localized basolaterally in FRCs in association with laminin *in vivo*. We find that LL5β basolateral localization is disrupted during LN expansion, which can account for the progressive loss of ECM during LN expansion.

Our results confirm that the FRC cellular network remains connected in inflamed LNs (Acton et al., 2014; Astarita et al., 2015), even though the conduit flow is locally disrupted. This also indicates that FRC-FRC connectivity is prioritized over maintaining ECM production. Our data suggest that to expand the LN rapidly, FRCs detach temporarily from the conduit and halt matrix production, leading to the local loss of conduit function as pre-existing ECM fibers are stretched through the expanding tissue. However, the remaining intact sections of conduit are sufficient to channel the lymph throughout the LN parenchyma. Recent observations based on FRC ablation and a graph theory-based systems biology approach have demonstrated that FRCs establish ''small-world'' networks (Novkovic et al., 2016; Textor et al., 2016). High local connectivity ensures the high topological robustness of the FRC network, which can tolerate the loss of 50% of all FRCs (Novkovic et al., 2016). We propose that a similar principle applies to conduit flow, in which interruption of the conduit network is efficiently overcome by sufficient alternative routes. These findings lead us to the question of how conduit flow is determined, whether as a result of pressure from tissues and afferent lymphatics pushing fluid through the conduit network or, alternatively, whether the draw of fluid leaving the system via high endothelial venules (HEVs) and efferent vessels provides a pulling force. Since we find no evidence for leakiness of conduits, which may be predicted in a model of pushing forces, our data may suggest that flow is determined by pull from the circulation. We cannot exclude that the dysfunctional conduit flow that we observe in many sections of the FRC network (Figure 6) is not due to a disruption or an alteration in the drainage of tissue fluid such that conduits do not fill properly.

In contexts outside the LN, PDPN is often upregulated by fibroblasts in inflammatory settings (Hisakane et al., 2016; Kim et al., 2015; Shindo et al., 2013). In these scenarios, the ECM deposition by these fibroblasts could also be regulated and modified by contact with CLEC-2$^+$ myeloid cells or platelets leaking from inflamed vessels. It will be interesting to understand whether the same CLEC-2-dependent transcriptional and protein expression regulation occurs in other PDPN$^+$ fibroblasts. Since the CLEC-2/PDPN signaling axis also regulates FRC actomyosin contractility, this signaling pathway may also control how ECM is aligned and organized in other tissues.

STAR★METHODS

Detailed methods are provided in the online version of this paper and include the following:

- KEY RESOURCES TABLE
- LEAD CONTACT AND MATERIALS AVAILABILITY
- EXPERIMENTAL MODEL AND SUBJECT DETAILS
  - Mice
  - Animal procedures
- METHOD DETAILS
  - Tissue clearing and immunostaining of intact LNs
  - Electron microscopy of LN conduits
  - Immunostaining of tissue sections
  - Image analysis
  - RNaseq analysis
  - FRC-derived matrices
  - Proteomics of FRC-derived matrices
  - Isobaric Tandem Mass Tag (TMT) Phosphoproteomics
  - GFP secretion assay
  - Western Blotting
  - Quantitative RT-PCR analysis for Phldb2 (LL5β) messenger RNA
  - Three-dimensional cell culture
  - Immunostaining of cells *in vitro*
  - LL5β silencing by siRNA
  - Flow cytometry of LNs
- QUANTIFICATION AND STATISTICAL ANALYSIS
- DATA AND CODE AVAILABILITY

SUPPLEMENTAL INFORMATION

Supplemental Information can be found online at https://doi.org/10.1016/j.celrep.2019.10.103.

ACKNOWLEDGMENTS

The authors are grateful to J. Sanes for the LL5β antibody, C. Reis e Sousa for the CD11c$^{\Delta CLEC\text{-}2}$ mice, A. Vaughan for assistance with microscopy, and C. Bennett for assistance with the *in vivo* experiments. They are also grateful to C. Bennett, C.M. de Winde, and E. Sahai for critical reading of the manuscript. This work is supported by a Cancer Research UK (CRUK-A19763) (to S.E.A.) and Medical Research Council grant MC_U12266B. C.J.T. is supported by Cancer Research UK (C60693/A23783). T.S. is supported by the Wellcome Trust (105604/Z/14/Z). S.D. is supported by the Swiss National Science Foundation (grants P2BSP3_158804 and P300PA_167657) and an EHA Research Grant award from the European Hematology Association. L.K. and P.H.H. are supported by the Institute of Cancer Research and Breast Cancer Now (2014NovPR360).

AUTHOR CONTRIBUTIONS

V.G.M. and S.E.A designed the study and wrote the original draft manuscript. V.G.M., V.P., and A.C.B. performed the *in vitro* experiments. V.G.M., S.M., and S.D. performed and analyzed the *in vivo* experiments. L.K. and P.H.H. performed the mass spectrometry of the ECM. C.J.T. designed and performed the TMT-proteomics experiments. I.J.W. and J.J.B. performed the electron microscopy (EM) of the conduit structures. T.S., H.L.H., and J.K.-V. performed the bioinformatics and statistical analyses. All of the authors contributed to

the data analysis and editing the manuscript. Funding was acquired by S.E.A., C.J.T., and P.H.H.

DECLARATION OF INTERESTS

The authors declare no competing interests.

Received: February 5, 2019
Revised: October 9, 2019
Accepted: October 25, 2019
Published: November 26, 2019

REFERENCES

Acton, S.E., Astarita, J.L., Malhotra, D., Lukacs-Kornek, V., Franz, B., Hess, P.R., Jakus, Z., Kuligowski, M., Fletcher, A.L., Elpek, K.G., et al. (2012). Podoplanin-rich stromal networks induce dendritic cell motility via activation of the C-type lectin receptor CLEC-2. Immunity *37*, 276–289.

Acton, S.E., Farrugia, A.J., Astarita, J.L., Mourão-Sá, D., Jenkins, R.P., Nye, E., Hooper, S., van Blijswijk, J., Rogers, N.C., Snelgrove, K.J., et al. (2014). Dendritic cells control fibroblastic reticular network tension and lymph node expansion. Nature *514*, 498–502.

Afratis, N.A., Nikitovic, D., Multhaupt, H.A.B., Theocharis, A.D., Couchman, J.R., and Karamanos, N.K. (2017). Syndecans - key regulators of cell signaling and biological functions. FEBS J. *284*, 27–41.

Astarita, J.L., Cremasco, V., Fu, J., Darnell, M.C., Peck, J.R., Nieves-Bonilla, J.M., Song, K., Kondo, Y., Woodruff, M.C., Gogineni, A., et al. (2015). The CLEC-2-podoplanin axis controls the contractility of fibroblastic reticular cells and lymph node microarchitecture. Nat. Immunol. *16*, 75–84.

Bajénoff, M., and Germain, R.N. (2009). B-cell follicle development remodels the conduit system and allows soluble antigen delivery to follicular dendritic cells. Blood *114*, 4989–4997.

Blagoveshchenskaya, A.D., Hannah, M.J., Allen, S., and Cutler, D.F. (2002). Selective and signal-dependent recruitment of membrane proteins to secretory granules formed by heterologously expressed von Willebrand factor. Mol. Biol. Cell *13*, 1582–1593.

Blum, R., Stephens, D.J., and Schulz, I. (2000). Lumenal targeted GFP, used as a marker of soluble cargo, visualises rapid ERGIC to Golgi traffic by a tubulo-vesicular network. J. Cell Sci. *113*, 3151–3159.

Bost, F., Diarra-Mehrpour, M., and Martin, J.-P. (1998). Inter-alpha-trypsin inhibitor proteoglycan family–a group of proteins binding and stabilizing the extracellular matrix. Eur. J. Biochem. *252*, 339–346.

Buechler, M.B., and Turley, S.J. (2018). A short field guide to fibroblast function in immunity. Semin. Immunol. *35*, 48–58.

Calvo, F., Ege, N., Grande-Garcia, A., Hooper, S., Jenkins, R.P., Chaudhry, S.I., Harrington, K., Williamson, P., Moeendarbary, E., Charras, G., and Sahai, E. (2013). Mechanotransduction and YAP-dependent matrix remodelling is required for the generation and maintenance of cancer-associated fibroblasts. Nat. Cell Biol. *15*, 637–646.

Casazza, A., Fazzari, P., and Tamagnone, L. (2007). Semaphorin signals in cell adhesion and cell migration: functional role and molecular mechanisms. Adv. Exp. Med. Biol. *600*, 90–108.

Castello, L.M., Raineri, D., Salmi, L., Clemente, N., Vaschetto, R., Quaglia, M., Garzaro, M., Gentilli, S., Navalesi, P., Cantaluppi, V., et al. (2017). Osteopontin at the Crossroads of Inflammation and Tumor Progression. Mediators Inflamm. *2017*, 4049098.

Cremasco, V., Woodruff, M.C., Onder, L., Cupovic, J., Nieves-Bonilla, J.M., Schildberg, F.A., Chang, J., Cremasco, F., Harvey, C.J., Wucherpfennig, K., et al. (2014). B cell homeostasis and follicle confines are governed by fibroblastic reticular cells. Nat. Immunol. *15*, 973–981.

Dancevic, C.M., Fraser, F.W., Smith, A.D., Stupka, N., Ward, A.C., and McCulloch, D.R. (2013). Biosynthesis and expression of a disintegrin-like and metalloproteinase domain with thrombospondin-1 repeats-15: a novel versican-cleaving proteoglycanase. J. Biol. Chem. *288*, 37267–37276.

de Vega, S., Hozumi, K., Suzuki, N., Nonaka, R., Seo, E., Takeda, A., Ikeuchi, T., Nomizu, M., Yamada, Y., and Arikawa-Hirasawa, E. (2016). Identification of peptides derived from the C-terminal domain of fibulin-7 active for endothelial cell adhesion and tube formation disruption. Biopolymers *106*, 184–195.

Dobin, A., Davis, C.A., Schlesinger, F., Drenkow, J., Zaleski, C., Jha, S., Batut, P., Chaisson, M., and Gingeras, T.R. (2013). STAR: ultrafast universal RNA-seq aligner. Bioinformatics *29*, 15–21.

Dubrot, J., Duraes, F.V., Potin, L., Capotosti, F., Brighouse, D., Suter, T., LeibundGut-Landmann, S., Garbi, N., Reith, W., Swartz, M.A., and Hugues, S. (2014). Lymph node stromal cells acquire peptide-MHCII complexes from dendritic cells and induce antigen-specific $CD4^+$ T cell tolerance. J. Exp. Med. *211*, 1153–1166.

Ellis, P.D., Metcalfe, J.C., Hyvönen, M., and Kemp, P.R. (2003). Adhesion of endothelial cells to NOV is mediated by the integrins alphavbeta3 and alpha5-beta1. J. Vasc. Res. *40*, 234–243.

Enomoto, Y., Matsushima, S., Shibata, K., Aoshima, Y., Yagi, H., Meguro, S., Kawasaki, H., Kosugi, I., Fujisawa, T., Enomoto, N., et al. (2018). LTBP2 is secreted from lung myofibroblasts and is a potential biomarker for idiopathic pulmonary fibrosis. Clin. Sci. (Lond.) *132*, 1565–1580.

Esnault, S., Torr, E.E., Bernau, K., Johansson, M.W., Kelly, E.A., Sandbo, N., and Jarjour, N.N. (2017). Endogenous Semaphorin-7A Impedes Human Lung Fibroblast Differentiation. PLoS One *12*, e0170207.

Evanko, S.P., Potter-Perigo, S., Bollyky, P.L., Nepom, G.T., and Wight, T.N. (2012). Hyaluronan and versican in the control of human T-lymphocyte adhesion and migration. Matrix Biol. *31*, 90–100.

Fletcher, A.L., Lukacs-Kornek, V., Reynoso, E.D., Pinner, S.E., Bellemare-Pelletier, A., Curry, M.S., Collier, A.-R., Boyd, R.L., and Turley, S.J. (2010). Lymph node fibroblastic reticular cells directly present peripheral tissue antigen under steady-state and inflammatory conditions. J. Exp. Med. *207*, 689–697.

Flevaris, P., and Vaughan, D. (2017). The Role of Plasminogen Activator Inhibitor Type-1 in Fibrosis. Semin. Thromb. Hemost. *43*, 169–177.

Franco-Barraza, J., Beacham, D.A., Amatangelo, M.D., and Cukierman, E. (2016). Preparation of Extracellular Matrices Produced by Cultured and Primary Fibroblasts. Curr. Protoc. Cell Biol. *71*, 10.9.1–10.9.34.

Gaggioli, C., Hooper, S., Hidalgo-Carcedo, C., Grosse, R., Marshall, J.F., Harrington, K., and Sahai, E. (2007). Fibroblast-led collective invasion of carcinoma cells with differing roles for RhoGTPases in leading and following cells. Nat. Cell Biol. *9*, 1392–1400.

Gagliardi, F., Narayanan, A., and Mortini, P. (2017). SPARCL1 a novel player in cancer biology. Crit. Rev. Oncol. Hematol. *109*, 63–68.

Gretz, J.E., Norbury, C.C., Anderson, A.O., Proudfoot, A.E., and Shaw, S. (2000). Lymph-borne chemokines and other low molecular weight molecules reach high endothelial venules via specialized conduits while a functional barrier limits access to the lymphocyte microenvironments in lymph node cortex. J. Exp. Med. *192*, 1425–1440.

Hamm, M.J., Kirchmaier, B.C., and Herzog, W. (2016). Sema3d controls collective endothelial cell migration by distinct mechanisms via Nrp1 and PlxnD1. J. Cell Biol. *215*, 415–430.

Harada, H., and Takahashi, M. (2007). CD44-dependent intracellular and extracellular catabolism of hyaluronic acid by hyaluronidase-1 and -2. J. Biol. Chem. *282*, 5597–5607.

Hisakane, K., Saruwatari, K., Fujii, S., Kirita, K., Umemura, S., Matsumoto, S., Yoh, K., Niho, S., Ohmatsu, H., Kuwata, T., et al. (2016). Unique intravascular tumor microenvironment predicting recurrence of lung squamous cell carcinoma. J. Cancer Res. Clin. Oncol. *142*, 593–600.

Hotta, A., Kawakatsu, T., Nakatani, T., Sato, T., Matsui, C., Sukezane, T., Akagi, T., Hamaji, T., Grigoriev, I., Akhmanova, A., et al. (2010). Laminin-based cell adhesion anchors microtubule plus ends to the epithelial cell basal cortex through LL5alpha/beta. J. Cell Biol. *189*, 901–917.

Jia, W., Li, H., and He, Y.-W. (2005). The extracellular matrix protein mindin serves as an integrin ligand and is critical for inflammatory cell recruitment. Blood *106*, 3854–3859.

Käll, L., Canterbury, J.D., Weston, J., Noble, W.S., and MacCoss, M.J. (2007). Semi-supervised learning for peptide identification from shotgun proteomics datasets. Nat. Methods *4*, 923–925.

Katoh, K., Kano, Y., Amano, M., Kaibuchi, K., and Fujiwara, K. (2001). Stress fiber organization regulated by MLCK and Rho-kinase in cultured human fibroblasts. Am. J. Physiol. Cell Physiol. *280*, C1669–C1679.

Kessler, E., Takahara, K., Biniaminov, L., Brusel, M., and Greenspan, D.S. (1996). Bone morphogenetic protein-1: the type I procollagen C-proteinase. Science *271*, 360–362.

Kim, H.-Y., Rha, K.-S., Shim, G.A., Kim, J.-H., Kim, J.-M., Huang, S.M., and Koo, B.S. (2015). Podoplanin is involved in the prognosis of head and neck squamous cell carcinoma through interaction with VEGF-C. Oncol. Rep. *34*, 833–842.

Krasny, L., Bland, P., Kogata, N., Wai, P., Howard, B.A., Natrajan, R.C., and Huang, P.H. (2018). SWATH mass spectrometry as a tool for quantitative profiling of the matrisome. J. Proteomics *189*, 11–22.

Lansbergen, G., Grigoriev, I., Mimori-Kiyosue, Y., Ohtsuka, T., Higa, S., Kitajima, I., Demmers, J., Galjart, N., Houtsmuller, A.B., Grosveld, F., and Akhmanova, A. (2006). CLASPs attach microtubule plus ends to the cell cortex through a complex with LL5beta. Dev. Cell *11*, 21–32.

Lee, J.-W., Epardaud, M., Sun, J., Becker, J.E., Cheng, A.C., Yonekura, A.-R., Heath, J.K., and Turley, S.J. (2007). Peripheral antigen display by lymph node stroma promotes T cell tolerance to intestinal self. Nat. Immunol. *8*, 181–190.

Lee, C.H., Shah, B., Moioli, E.K., and Mao, J.J. (2010). CTGF directs fibroblast differentiation from human mesenchymal stem/stromal cells and defines connective tissue healing in a rodent injury model. J. Clin. Invest. *120*, 3340–3349.

Link, A., Vogt, T.K., Favre, S., Britschgi, M.R., Acha-Orbea, H., Hinz, B., Cyster, J.G., and Luther, S.A. (2007). Fibroblastic reticular cells in lymph nodes regulate the homeostasis of naive T cells. Nat. Immunol. *8*, 1255–1265.

Malhotra, D., Fletcher, A.L., Astarita, J., Lukacs-Kornek, V., Tayalia, P., Gonzalez, S.F., Elpek, K.G., Chang, S.K., Knoblich, K., Hemler, M.E., et al.; Immunological Genome Project Consortium (2012). Transcriptional profiling of stroma from inflamed and resting lymph nodes defines immunological hallmarks. Nat. Immunol. *13*, 499–510.

McDowell, G.S., Gaun, A., and Steen, H. (2013). iFASP: combining isobaric mass tagging with filter-aided sample preparation. J. Proteome Res. *12*, 3809–3812.

Mi, H., Muruganujan, A., Casagrande, J.T., and Thomas, P.D. (2013). Large-scale gene function analysis with the PANTHER classification system. Nat. Protoc. *8*, 1551–1566.

Mi, H., Huang, X., Muruganujan, A., Tang, H., Mills, C., Kang, D., and Thomas, P.D. (2017). PANTHER version 11: expanded annotation data from Gene Ontology and Reactome pathways, and data analysis tool enhancements. Nucleic Acids Res. *45* (*D1*), D183–D189.

Murdamoothoo, D., Schwenzer, A., Kant, J., Rupp, T., Marzeda, A., Midwood, K., and Orend, G. (2018). Investigating cell-type specific functions of tenascin-C. Methods Cell Biol. *143*, 401–428.

Naba, A., Clauser, K.R., Hoersch, S., Liu, H., Carr, S.A., and Hynes, R.O. (2012). The matrisome: in silico definition and in vivo characterization by proteomics of normal and tumor extracellular matrices. Mol. Cell. Proteomics *11*, M111.014647.

Naba, A., Clauser, K.R., Ding, H., Whittaker, C.A., Carr, S.A., and Hynes, R.O. (2016). The extracellular matrix: Tools and insights for the "omics" era. Matrix Biol. *49*, 10–24.

Naba, A., Pearce, O.M.T., Del Rosario, A., Ma, D., Ding, H., Rajeeve, V., Cutillas, P.R., Balkwill, F.R., and Hynes, R.O. (2017). Characterization of the Extracellular Matrix of Normal and Diseased Tissue Using Proteomics. J. Proteome Res. *16*, 3083–3091.

Nagamine, S., Tamba, M., Ishimine, H., Araki, K., Shiomi, K., Okada, T., Ohto, T., Kunita, S., Takahashi, S., Wismans, R.G.P., et al. (2012). Organ-specific sulfation patterns of heparan sulfate generated by extracellular sulfatases Sulf1 and Sulf2 in mice. J. Biol. Chem. *287*, 9579–9590.

Niimori, D., Kawano, R., Niimori-Kita, K., Ihn, H., and Ohta, K. (2014). Tsukushi is involved in the wound healing by regulating the expression of cytokines and growth factors. J. Cell Commun. Signal. *8*, 173–177.

Nolte, M.A., Beliën, J.A.M., Schadee-Eestermans, I., Jansen, W., Unger, W.W.J., van Rooijen, N., Kraal, G., and Mebius, R.E. (2003). A conduit system distributes chemokines and small blood-borne molecules through the splenic white pulp. J. Exp. Med. *198*, 505–512.

Noordstra, I., and Akhmanova, A. (2017). Linking cortical microtubule attachment and exocytosis. F1000Res. *6*, 469.

Novkovic, M., Onder, L., Cupovic, J., Abe, J., Bomze, D., Cremasco, V., Scandella, E., Stein, J.V., Bocharov, G., Turley, S.J., and Ludewig, B. (2016). Topological Small-World Organization of the Fibroblastic Reticular Cell Network Determines Lymph Node Functionality. PLoS Biol. *14*, e1002515.

Novkovic, M., Onder, L., Cheng, H.-W., Bocharov, G., and Ludewig, B. (2018). Integrative Computational Modeling of the Lymph Node Stromal Cell Landscape. Front. Immunol. *9*, 2428.

Obenauer, J.C., Cantley, L.C., and Yaffe, M.B. (2003). Scansite 2.0: proteome-wide prediction of cell signaling interactions using short sequence motifs. Nucleic Acids Res. *31*, 3635–3641.

Ohashi, K., Enomoto, T., Joki, Y., Shibata, R., Ogura, Y., Kataoka, Y., Shimizu, Y., Kambara, T., Uemura, Y., Yuasa, D., et al. (2014). Neuron-derived neurotrophic factor functions as a novel modulator that enhances endothelial cell function and revascularization processes. J. Biol. Chem. *289*, 14132–14144.

Ono, M., Masaki, A., Maeda, A., Kilts, T.M., Hara, E.S., Komori, T., Pham, H., Kuboki, T., and Young, M.F. (2018). CCN4/WISP1 controls cutaneous wound healing by modulating proliferation, migration and ECM expression in dermal fibroblasts via $\alpha 5\beta 1$ and TNFα. Matrix Biol. *68-69*, 533–546.

Palframan, R.T., Jung, S., Cheng, G., Weninger, W., Luo, Y., Dorf, M., Littman, D.R., Rollins, B.J., Zweerink, H., Rot, A., and von Andrian, U.H. (2001). Inflammatory chemokine transport and presentation in HEV: a remote control mechanism for monocyte recruitment to lymph nodes in inflamed tissues. J. Exp. Med. *194*, 1361–1373.

Patro, R., Duggal, G., Love, M.I., Irizarry, R.A., and Kingsford, C. (2017). Salmon provides fast and bias-aware quantification of transcript expression. Nat. Methods *14*, 417–419.

Porter, S., Clark, I.M., Kevorkian, L., and Edwards, D.R. (2005). The ADAMTS metalloproteinases. Biochem. J. *386*, 15–27.

Qadri, M.M., Jay, G.D., Ostrom, R.S., Zhang, L.X., and Elsaid, K.A. (2018). cAMP attenuates TGF-β's profibrotic responses in osteoarthritic synoviocytes: involvement of hyaluronan and PRG4. Am. J. Physiol. Cell Physiol. *315*, C432–C443.

Radtke, A.J., Kastenmüller, W., Espinosa, D.A., Gerner, M.Y., Tse, S.-W., Sinnis, P., Germain, R.N., Zavala, F.P., and Cockburn, I.A. (2015). Lymph-node resident CD8α+ dendritic cells capture antigens from migratory malaria sporozoites and induce CD8+ T cell responses. PLoS Pathog. *11*, e1004637.

Randolph, G.J., Ivanov, S., Zinselmeyer, B.H., and Scallan, J.P. (2017). The Lymphatic System: Integral Roles in Immunity. Annu. Rev. Immunol. *35*, 31–52.

Rantakari, P., Auvinen, K., Jäppinen, N., Kapraali, M., Valtonen, J., Karikoski, M., Gerke, H., Iftakhar-E-Khuda, I., Keuschnigg, J., Umemoto, E., et al. (2015). The endothelial protein PLVAP in lymphatics controls the entry of lymphocytes and antigens into lymph nodes. Nat. Immunol. *16*, 386–396.

Riedel, A., Shorthouse, D., Haas, L., Hall, B.A., and Shields, J. (2016). Tumor-induced stromal reprogramming drives lymph node transformation. Nat. Immunol. *17*, 1118–1127.

Riessen, R., Fenchel, M., Chen, H., Axel, D.I., Karsch, K.R., and Lawler, J. (2001). Cartilage oligomeric matrix protein (thrombospondin-5) is expressed by human vascular smooth muscle cells. Arterioscler. Thromb. Vasc. Biol. *21*, 47–54.

Rodda, L.B., Lu, E., Bennett, M.L., Sokol, C.L., Wang, X., Luther, S.A., Barres, B.A., Luster, A.D., Ye, C.J., and Cyster, J.G. (2018). Single-Cell RNA Sequencing of Lymph Node Stromal Cells Reveals Niche-Associated Heterogeneity. Immunity *48*, 1–29.

Rodrigues-Ferreira, S., Abdelkarim, M., Dillenburg-Pilla, P., Luissint, A.-C., di-Tommaso, A., Deshayes, F., Pontes, C.L.S., Molina, A., Cagnard, N., Letourneur, F., et al. (2012). Angiotensin II facilitates breast cancer cell migration and metastasis. PLoS One *7*, e35667.

Rohner, N.A., McClain, J., Tuell, S.L., Warner, A., Smith, B., Yun, Y., Mohan, A., Sushnitha, M., and Thomas, S.N. (2015). Lymph node biophysical remodeling is associated with melanoma lymphatic drainage. FASEB J. *29*, 4512–4522.

Roozendaal, R., Mempel, T.R., Pitcher, L.A., Gonzalez, S.F., Verschoor, A., Mebius, R.E., von Andrian, U.H., and Carroll, M.C. (2009). Conduits mediate transport of low-molecular-weight antigen to lymph node follicles. Immunity *30*, 264–276.

Roth, L., Kalev-Altman, R., Monsonego-Ornan, E., and Sela-Donenfeld, D. (2017). A new role of the membrane-type matrix metalloproteinase 16 (MMP16/MT3-MMP) in neural crest cell migration. Int. J. Dev. Biol. *61*, 245–256.

Roychaudhuri, R., Hergrueter, A.H., Polverino, F., Laucho-Contreras, M.E., Gupta, K., Borregaard, N., and Owen, C.A. (2014). ADAM9 is a novel product of polymorphonuclear neutrophils: regulation of expression and contributions to extracellular matrix protein degradation during acute lung injury. J.I Immunol. *193*, 2469–2482.

Schacker, T.W., Brenchley, J.M., Beilman, G.J., Reilly, C., Pambuccian, S.E., Taylor, J., Skarda, D., Larson, M., Douek, D.C., and Haase, A.T. (2006). Lymphatic tissue fibrosis is associated with reduced numbers of naive CD4+ T cells in human immunodeficiency virus type 1 infection. Clin. Vaccine Immunol. *13*, 556–560.

Seher, A., Nickel, J., Mueller, T.D., Kneitz, S., Gebhardt, S., ter Vehn, T.M., Schlunck, G., and Sebald, W. (2011). Gene expression profiling of connective tissue growth factor (CTGF) stimulated primary human tenon fibroblasts reveals an inflammatory and wound healing response in vitro. Mol. Vis. *17*, 53–62.

Shevchenko, A., Tomas, H., Havlis, J., Olsen, J.V., and Mann, M. (2006). In-gel digestion for mass spectrometric characterization of proteins and proteomes. Nat. Protoc. *1*, 2856–2860.

Shindo, K., Aishima, S., Ohuchida, K., Fujiwara, K., Fujino, M., Mizuuchi, Y., Hattori, M., Mizumoto, K., Tanaka, M., and Oda, Y. (2013). Podoplanin expression in cancer-associated fibroblasts enhances tumor progression of invasive ductal carcinoma of the pancreas. Mol. Cancer *12*, 168.

Sixt, M., Kanazawa, N., Selg, M., Samson, T., Roos, G., Reinhardt, D.P., Pabst, R., Lutz, M.B., and Sorokin, L. (2005). The conduit system transports soluble antigens from the afferent lymph to resident dendritic cells in the T cell area of the lymph node. Immunity *22*, 19–29.

Sobocinski, G.P., Toy, K., Bobrowski, W.F., Shaw, S., Anderson, A.O., and Kaldjian, E.P. (2010). Ultrastructural localization of extracellular matrix proteins of the lymph node cortex: evidence supporting the reticular network as a pathway for lymphocyte migration. BMC Immunol. *11*, 42.

Soneson, C., Love, M.I., and Robinson, M.D. (2015). Differential analyses for RNA-seq: transcript-level estimates improve gene-level inferences. F1000Res. *4*, 1521.

Song, E.-L., Hou, Y.-P., Yu, S.-P., Chen, S.-G., Huang, J.-T., Luo, T., Kong, L.-P., Xu, J., and Wang, H.-Q. (2011). EFEMP1 expression promotes angiogenesis and accelerates the growth of cervical cancer in vivo. Gynecol. Oncol. *121*, 174–180.

Stehbens, S.J., Paszek, M., Pemble, H., Ettinger, A., Gierke, S., and Wittmann, T. (2014). CLASPs link focal-adhesion-associated microtubule capture to localized exocytosis and adhesion site turnover. Nat. Cell Biol. *16*, 561–573.

Sureshbabu, A., Okajima, H., Yamanaka, D., Tonner, E., Shastri, S., Maycock, J., Szymanowska, M., Shand, J., Takahashi, S., Beattie, J., et al. (2012). IGFBP5 induces cell adhesion, increases cell survival and inhibits cell migration in MCF-7 human breast cancer cells. J. Cell Sci. *125*, 1693–1705.

Tape, C.J., Worboys, J.D., Sinclair, J., Gourlay, R., Vogt, J., McMahon, K.M., Trost, M., Lauffenburger, D.A., Lamont, D.J., and Jørgensen, C. (2014). Reproducible automated phosphopeptide enrichment using magnetic TiO2 and Ti-IMAC. Anal. Chem. *86*, 10296–10302.

Tape, C.J., Ling, S., Dimitriadi, M., McMahon, K.M., Worboys, J.D., Leong, H.S., Norrie, I.C., Miller, C.J., Poulogiannis, G., Lauffenburger, D.A., and Jørgensen, C. (2016). Oncogenic KRAS Regulates Tumor Cell Signaling via Stromal Reciprocation. Cell *165*, 1818.

Taus, T., Köcher, T., Pichler, P., Paschke, C., Schmidt, A., Henrich, C., and Mechtler, K. (2011). Universal and confident phosphorylation site localization using phosphoRS. J. Proteome Res. *10*, 5354–5362.

Textor, J., Mandl, J.N., and de Boer, R.J. (2016). The Reticular Cell Network: A Robust Backbone for Immune Responses. PLoS Biol. *14*, e2000827.

Tocharus, J., Tsuchiya, A., Kajikawa, M., Ueta, Y., Oka, C., and Kawaichi, M. (2004). Developmentally regulated expression of mouse HtrA3 and its role as an inhibitor of TGF-beta signaling. Dev. Growth Differ. *46*, 257–274.

Tricaud, N. (2018). Myelinating Schwann Cell Polarity and Mechanically-Driven Myelin Sheath Elongation. Front. Cell. Neurosci. *11*, 414.

Wang, Z.-Q., Xing, W.-M., Fan, H.-H., Wang, K.-S., Zhang, H.-K., Wang, Q.-W., Qi, J., Yang, H.-M., Yang, J., Ren, Y.-N., et al. (2009). The novel lipopolysaccharide-binding protein CRISPLD2 is a critical serum protein to regulate endotoxin function. J.I Immunol. *183*, 6646–6656.

Wiśniewski, J.R., Zougman, A., Nagaraj, N., and Mann, M. (2009). Universal sample preparation method for proteome analysis. Nat. Methods *6*, 359–362.

Wynn, T.A., and Ramalingam, T.R. (2012). Mechanisms of fibrosis: therapeutic translation for fibrotic disease. Nat. Med. *18*, 1028–1040.

Xue, M., and Jackson, C.J. (2015). Extracellular Matrix Reorganization During Wound Healing and Its Impact on Abnormal Scarring. Adv. Wound Care (New Rochelle) *4*, 119–136.

Yang, B., Treweek, J.B., Kulkarni, R.P., Deverman, B.E., Chen, C.-K., Lubeck, E., Shah, S., Cai, L., and Gradinaru, V. (2014). Single-cell phenotyping within transparent intact tissue through whole-body clearing. Cell *158*, 945–958.

Yin, K., Shang, M., Dang, S., Wang, L., Xia, Y., Cui, L., Fan, X., Qu, J., Chen, J., and Xu, Z. (2018). Netrin-1 induces the proliferation of gastric cancer cells via the ERK/MAPK signaling pathway and FAK activation. Oncol. Rep. *40*, 2325–2333.

Yoshina, S., Sakaki, K., Yonezumi-Hayashi, A., Gengyo-Ando, K., Inoue, H., Iino, Y., and Mitani, S. (2012). Identification of a novel ADAMTS9/GON-1 function for protein transport from the ER to the Golgi. Mol. Biol. Cell *23*, 1728–1741.

Zeng, M., Smith, A.J., Wietgrefe, S.W., Southern, P.J., Schacker, T.W., Reilly, C.S., Estes, J.D., Burton, G.F., Silvestri, G., Lifson, J.D., et al. (2011). Cumulative mechanisms of lymphoid tissue fibrosis and T cell depletion in HIV-1 and SIV infections. J. Clin. Invest. *121*, 998–1008.

Zhai, H., Qi, X., Li, Z., Zhang, W., Li, C., Ji, L., Xu, K., and Zhong, H. (2018). TIMP-3 suppresses the proliferation and migration of SMCs from the aortic neck of atherosclerotic AAA in rabbits, via decreased MMP-2 and MMP-9 activity, and reduced TNF-α expression. Mol. Med. Rep. *18*, 2061–2067.

STAR★METHODS

KEY RESOURCES TABLE

REAGENT or RESOURCE	SOURCE	IDENTIFIER
Antibodies		
a-Tubulin	Thermo Fisher	A11126
CD11b	Biolegend	101225
CD11c	Biolegend	117333
Collagen I	Abcam	Ab34710
Collagen IV	Abcam	Ab6588
Fibronectin	Sigma-Aldrich	F3648
F4/80	Biolegend	123125
GFP	BioRad	47451051
I-A/I-E	Biolegend	107617
Phospho-paxillin	Cell signaling techology	2541S
Podoplanin	Acris	DM3501
Laminin	Abcam	Ab11575
LL5beta	Prof J. Sanes	N/A
Chemicals, Peptides, and Recombinant Proteins		
CLEC-2-Fc recombinant protein	Acton Lab	https://doi.org/10.1038/nature13814
Deposited Data		
RNaseq data CLEC-2/PDPN signaling in FRCs	This paper	https://doi.org/10.5522/04/c.4696979
CLEC-2/PDPN signaling phosphoproteomics	This paper	https://doi.org/10.5522/04/c.4696979
Proteomics of matrix production	This paper	https://doi.org/10.5522/04/c.4696979
Experimental Models: Cell Lines		
Immortalized FRCs	Acton Lab	https://doi.org/10.1038/nature13814
Immortalized FRCs – PDPN KD	Acton Lab	https://doi.org/10.1038/nature13814
Immortalized FRCs – CLEC-2 expressing	This paper	N/A
Experimental Models: Organisms/strains		
B6.129S4-Pdgfratm11(EGFP)Sor/J	Jackson	007669
CD11c$^{\Delta CLEC\text{-}2}$	Francis Crick Institute	https://doi.org/10.1038/nature13814
Oligonucleotides		
LL5beta siRNA	Dharmacon	LQ-062938-01
Software and Algorithms		
STAR aligner software	Dobin et al., 2013	https://github.com/alexdobin/STAR
Salmon software	Patro et al., 2017	https://combine-lab.github.io/salmon/
Bioconductor tximport package	Soneson et al., 2015	https://doi.org/10.18129/B9.bioc.tximport

LEAD CONTACT AND MATERIALS AVAILABILITY

Further information and requests for resources and reagents should be directed to and will be fulfilled by the Lead Contact, Sophie E. Acton (s.acton@ucl.ac.uk).

Key resources including details of key reagents and cell lines used and generated are available in the Key Resources Table. All unique/stable reagents generated in this study are available from the Lead Contact with a completed Materials Transfer Agreement.

EXPERIMENTAL MODEL AND SUBJECT DETAILS

Mice

Experiments were performed in accordance with national and institutional guidelines for animal care and approved by the Institutional Animal Ethics Committee Review Board, Cancer Research UK and the UK Home Office. Wild-type C57BL/6J mice were purchased from Charles River Laboratories. PDGFRaKI-H2BGFP mice (B6.129S4-Pdgfratm11(EGFP)Sor/J) were purchased from Jackson Laboratories. Generation of CD11c$^{\Delta CLEC\text{-}2}$ was achieved as previously described (Acton et al., 2014) by crossing Clec1b$^{fl/fl}$ with CD11c-Cre mice (B6.Cg-Tg(Itgax-cre)1.1Reiz). Both males and females were used for *in vivo* and *in vitro* experiments and were aged 8–12 weeks. Cre-negative littermates were used as controls in all experiments.

Animal procedures

Immunisations

Mice were immunized via subcutaneous injection in the right flank of 100 μL of an emulsion of OVA in CFA or IFA (100 μg OVA per mouse) (Hooke Laboratories). After 5 days, mice were culled and inguinal LNs from both flanks (naive and inflamed) were extracted for paired histological studies or flow cytometry analysis.

Dextran uptake in vivo

Five days after immunization, mice were injected subcutaneously in both flanks with 20 μL of dextran solution (100 μg dextran per flank) conjugated to: Cascade Blue (10kDa dextran), Tetramethylrhodamine (70 kDa dextran) or Fluorescein (500 kDa dextran), all from Thermo Fisher Scientific. Mice were culled and paired inguinal LNs (inflamed versus non-inflamed) collected after 30 or 90 minutes for histological and flow cytometry analysis respectively.

Cell lines

In vitro experiments were performed using immortalized WT (control) and PDPN knockdown mouse FRC cell lines previously described (Acton et al., 2014). For CLEC-2-Fc expression by FRCs, *Clec1b* cDNA was cloned into pFUSE-rIgG-Fc2 plasmids (Invivogen) and transfected into WT FRCs using lipofectamine 2000 (Thermo Fisher Scientific). Transfected cells were selected by prolonged culture with zeocin 100 μg/ml (Invivogen) and secretion of CLEC-2-Fc was confirmed by western blotting for cell-derived supernatants (data not shown).

FRC cell lines were cultured in DMEM plus glutamax (Life Technologies, Invitrogen) supplemented with 10% FBS, Penicillin-Streptomycin (100 U/mL) and 1% Insulin-Transferrin-Selenium (Life Technologies, Invitrogen) at 37°C in 10% CO_2. Cells were passaged when they reached 80%–90% confluence, by incubating in Cell Dissociation Buffer (Thermo Fisher Scientific) for 10 minutes at 37°C, plus a gentle treatment of 1 min with Trypsin 0.25% (Thermo Fisher Scientific). When indicated, FRCs were treated with 50 μg/ml CLEC-2-Fc or 10 μM ROCK inhibitor Y-27632 dihydrochloride (Tocris) for the last 2 hr of culture.

Primary cultures

Bone marrow cells were obtained from tibias and femurs from CD11c$^{\Delta CLEC\text{-}2}$ mice and Cre-negative control littermates. Whole bone marrow was cultured in non-treated 10 cm Petri dishes in RPMI media supplemented with 10% FBS and Penicillin-Streptomycin (100 U/mL) plus 20 ng/ml of recombinant murine GM-CSF (Peprotech) at 4x10^6 cells / 13 mL of medium. After 3 days, cultures were supplemented with 4 mL of fresh media plus 37.2 ng/ml GM-CSF. After 6 days in culture, BMDCs were stimulated with 10 ng/ml Lipopolysaccharides from *Escherichia coli* 0111:B4 (Sigma-Aldrich) for 24 hr before harvesting.

METHOD DETAILS

Tissue clearing and immunostaining of intact LNs

We used a modified version of the PACT (passive clarity technique) for whole LN staining based on previous publication (Yang et al., 2014). In brief, AntigenFix (DiaPath) fixed LNs were incubated overnight at 4°C in 40% acrylamide + 25 mg/ml 2,2′-Azobis(2-methylpropionamidine) dihydrochloride (Sigma). Infused samples were degassed with nitrogen for 5 min and then incubated for 6 hr at 37°C. Samples were washed in PBS for 24 hr and incubated for 4 days with 8% SDS PBS solution at 37°C. After washing with PBS for 24 hr, LNs were incubated with 1:100 anti-collagen IV in PBS 2% goat serum (Abcam) 0.1% Triton X-100 (Sigma-Aldrich) 0.01% sodium azide (Webscientific) for 4 days at 37°C (rotation). Same concentration of the antibody was added after 1 and 2 days. Then wash in PBS at 37°C (rotation) for 24 hr and incubated with the secondary antibody in same conditions as the primary. Samples were transferred to a 2 g/ml Histodenz (Sigma-Aldricht), 0.01% sodium azide solution in PBS, incubated for 24 hr and imaged in this medium. Imaging was performed on a Leica TCS SP8 STED 3X using HC FLUOTAR L VISIR 25x water lenses.

Electron microscopy of LN conduits

LNs were fixed overnight in 2% PFA/1.5% glutaraldehyde (both EM grade from TAAB) in 0.1M sodium Cacodylate at 4°C and embedded in 2.8% low melting point agarose dissolved in PBS. Slices of 100 μm thickness were then cut in cold PBS using a vibrating microtome (VT1200S; Leica) and returned to fresh fix solution for a further 15 mins. Slices were then secondarily fixed for 1 h in 1% osmium tetraoxide/1.5% potassium ferricyanide at 4°C and then treated with 1% tannic acid in 0.1M sodium cacodylate for 45min at room temperature. Samples were then dehydrated in sequentially increasing concentration of ethanol solutions, and embedded in Epon resin. The 70nm ultrathin resin sections were cut with a Diatome 45° diamond knife using an ultramicrotome

(UC7; Leica). Sections were collected on 1 × 2mm formvar-coated slot grids and stained with Reynolds lead citrate. All samples were imaged using a transmission electron microscope (Tecnai T12; FEI) equipped with a charge-coupled device camera (SIS Morada; Olympus).

Immunostaining of tissue sections

LN samples were fixed in AntigenFix (DiaPath) overnight, washed and incubated in PBS 30% sucrose (w/v) (Sigma-Aldrich) overnight at 4°C. Samples were embedded in Tissue-Tek® O.C.T. Compound (Thomas Scientific) and frozen using 2-Methylbutane, cooled with liquid nitrogen. 20 μm sections were cut using a Leica CM1850 cryostat. For immunostaining, tissue sections were blocked for 2 hr at room temperature in 10% goat normal Serum (Sigma-Aldrich), 0.3% Triton X-100 (Sigma-Aldrich) in PBS. Primary antibodies were incubated overnight at 4°C in 10% goat normal Serum (Sigma-Aldrich), 0.01% Triton X-100 (Sigma-Aldrich) in PBS. 3 washing steps were used to remove unbound antibody, before incubation with the secondary antibody plus Hoechst (Fisher Scientific) for 2 hr at room temperature. Samples were washed and mounted in mowiol. Samples were imaged on a Leica TCS SP8 STED 3X using HC PL APO CS2 /1.4 63x oil lenses.

Image analysis

Conduit components

Podoplanin staining was used to define the FRC network in LN frozen tissue sections. Podoplanin signal was filtered by Gaussian Blur (sigma = 2) to remove background and thresholded identically in all samples. We next created a selection that was used in the corresponding channels in order to obtain the median intensity of the conduit components. We performed this process in a number of regions of interest within the T cell area for each LN, always minimizing the presence of vasculature.

Semiautomated quantification of focal adhesion length

Signal for phospho-Paxillin staining was thresholded equally in all samples after removal of background noise by Gaussian Blur (sigma = 2). Focal adhesions were segmented using the analyze particle tool in Fiji and fit ellipse. Major axis of the ellipse was used as an estimation of focal adhesion length.

RNaseq analysis

FRCs were cultured for 24h, adding 50 μg/ml of CLEC-2-Fc from then beginning or 6 hours before collecting cells. Cells were left untreated as a control. RNA extractions were performed using the RNAeasy kit (QIAGEN) following the manufacturer's instructions, including a DNA digestion step to avoid genome contamination in further analysis.

For transcriptome sequencing and analysis, RNA preparations from FRCs were sequenced to a depth of 9 million to 22 million reads by Queen Mary University (QMUL) Genome Centre. The raw read quality control was performed by the QMUL Genome Centre using BaseSpace Illumina software. Paired end FASTQ files were then aligned to *Mus musculus* GRCm38 reference assembly using STAR aligner software (Dobin et al., 2013). Transcripts were assembled and relative transcript abundance were calculated using Salmon software (Patro et al., 2017). Using R (v3.4.4) and the Bioconductor tximport package (Soneson et al., 2015), TPM (Transcripts per million) values were generated and annotated with ENSEMBL gene IDs. Bulk TPM data were categorised by the fold change (> 2 fold) between control, 6 hr and 24 hr conditions using an in-house developed R script. Gene Ontology analysis were performed using the PANTHER software (Mi et al., 2017; 2013) and PCA plots were generated using the ggplot package in R.

FRC-derived matrices

FRC-derived matrices were generated *in vitro* according to published methods (Franco-Barraza et al., 2016). In brief, gelatin-coated wells were used to culture FRC cell lines at $5x10^3$ cells/cm^2 in culture media supplemented with 50 μg/ml L(+)-Ascorbic acid sodium salt (Sigma-Aldrich) for 5 days, unless otherwise stated. Supplemented media was replenished at day 1 and 3. For proteomic analysis, cells in their matrix were collected in PBS, centrifuged and resuspended in 4M Urea. For microscopy analysis, cells were lysed incubating for 15 min at 37°C in PBS 1% Triton X-100 20 mM ammonium hydroxide. Matrices were blocked with 2% bovine serum albumin (w/v) (Sigma-Aldrich) and stained with the indicated antibodies. Samples were imaged with a Leica TCS SP5 Confocal Microscope using 63X oil HCX PL APO lenses.

Proteomics of FRC-derived matrices

Quantitative proteomic analysis of the FRC-derived matrices was performed by sequential window acquisition of all theoretical spectra mass spectrometry (SWATH MS). For construction of the spectral library, FRCs and derived matrices were washed in PBS, centrifuged and enriched for extracellular matrix as previously described in Krasny et al. (Krasny et al., 2018). Enriched matrices were digested using gel-assisted protocol (Shevchenko et al., 2006) and desalted prior analysis by liquid chromatography-tandem mass spectrometry (LC-MS/MS) on Agilent 1260 HPLC coupled to TripleTOF 5600+ (SCIEX) mass spectrometer in data-dependent acquisition mode. For LC-MS/MS, peptides were spiked with iRT peptides (Biognosys AG), loaded on a 75 μm x 15 cm long analytical column packed with Reprosil Pur C18-AQ 3 μm resin (Dr Maisch) end eluted using a linear gradient of 2%–40% of Buffer B (98% ACN, 0.1% FA) in 90 min at flow rate of 250nl/min. Acquired datasets were searched by ProteinPilot 5.0.1 software (Sciex) against a Swissprot mouse database and spectral library was generated in Spectronaut 11 (Biognosys AG) from the results and combined with previously published library (Krasny et al., 2018). For quantitative analysis, FRCs and derived matrices were lysed on ice in

8M Urea, 100mM ammonium bicarbonate buffer and digested using gel-assisted protocol. Desalted peptides were spiked with iRT peptides and analyzed on the same LC-MS/MS instrument using identical LC conditions. MS/MS data were acquired in 60 SWATH windows with fixed size of 13 Da. SWATH spectra were analyzed in Spectronaut 11 with FDR restricted to 1%. Further statistical processing of median normalized data was performed in Perseus (1.5.6) [91].

Isobaric Tandem Mass Tag (TMT) Phosphoproteomics

Isobaric Tandem Mass Tag (TMT) Phosphoproteomics were performed as described in Tape et al. (2016) (PMID: 27087446). Following treatment, FRCs were lysed in 6 M urea, 10 mM NaPPi, 20 mM HEPES, pH 8.0, sonicated, centrifuged to clear cell debris, and protein concentration was determined by BCA (Pierce 23225). 200 μg of each condition were individually digested by FASP (Wiśniewski et al., 2009), amine-TMT-10-plex-labeled (Pierce 90111) on membrane (iFASP) (McDowell et al., 2013), eluted, pooled, lyophilized, and subjected to automated phosphopeptide enrichment (APE) (Tape et al., 2014). Phosphopeptides were desalted using OLIGO R3 resin (Life Technologies 1-1339-03) and lyophilized prior to liquid chromatography-tandem mass spectrometry (LC-MS/MS) analysis. Samples were run on a Q-Exactive Plus mass spectrometer (Thermo Scientific) coupled to a Dionex Ultimate 3000 RSLC nano system (Thermo Scientific). Reversed-phase chromatographic separation was performed on a C18 PepMap 300 A trap cartridge (0.3 mm i.d. x 5 mm, 5 μm bead size; loaded in a bi-directional manner), a 75 μm i.d. x 50 cm column (5 μm bead size) using a 120 minute linear gradient of 0%–50% solvent B (MeCN 100% + 0.1% formic acid (FA)) against solvent A (H2O 100% + 0.1% FA) with a flow rate of 300 nL/min. The mass spectrometer was operated in the data-dependent mode to automatically switch between Orbitrap MS and MS/MS acquisition. Survey full scan MS spectra (from m/z 400-2000) were acquired in the Orbitrap with a resolution of 70,000 at m/z 400 and FT target value of 1×10^6 ions. The 20 most abundant ions were selected for fragmentation using higher-energy collisional dissociation (HCD) and dynamically excluded for 30 s. Fragmented ions were scanned in the Orbitrap at a resolution of 35,000 (TMT) at m/z 400. The isolation window was reduced to 1.2 m/z and a MS/MS fixed first mass of 120 m/z was used to aid TMT detection. For accurate mass measurement, the lock mass option was enabled using the polydimethylcyclosiloxane ion (m/z 445.120025) as an internal calibrant. For peptide identification, raw data files produced in Xcalibur 2.1 (Thermo Scientific) were processed in Proteome Discoverer 1.4 (Thermo Scientific) and searched against SwissProt mouse (2011_03 release, 15,082,690 entries) database using Mascot (v2.2). Searches were performed with a precursor mass tolerance set to 10 ppm, fragment mass tolerance set to 0.05 Da and a maximum number of missed cleavages set to 2. Peptides were further filtered using a mascot significance threshold < 0.05, a peptide ion Score > 20 and a FDR < 0.01 (evaluated by Percolator (Käll et al., 2007). Phospho-site localization probabilities were calculated with phosphoRS 3.1 (> 75%, maximum 4-PTM/peptide) (Taus et al., 2011). Phosphopeptides from Proteome Discoverer 1.4 were normalized against total protein levels (from in-gel digest experiments), and protein-level phospho-site locations (phosphoRS 3.1 score > 75%, maximum 4-PTM/peptide) were manually annotated using PhosphoSitePlus. Phosphoproteomic volcano plots display mean Proteome Discoverer 1.4 quantification fold-difference values across all replicates (log2) against two-tailed t test P values (calculated from arrays of raw MS/MS TMT intensity counts). Volcano plots were assembled in GraphPad Prism 6 (non-linear Gaussian regression, least-squares fit). For principle component analysis (PCA), Proteome Discoverer 1.4 quantification ratio values were converted to log2, imported into R (version 3.0.1), computed using the function ‘princomp(X)’ and plotted in GraphPad Prism. Empirical parent kinases were manually identified by referenced Uniprot annotation and putative parent kinases were manually assigned using ScanSite (Obenauer et al., 2003) 3 (‘High- Stringency’ setting, top 0.2% of all sites, lowest score). Phospho-sites that did not meet these conditions were not annotated.

GFP secretion assay

FRC cells lines were transfected with 500 ng of lumGFP plasmid (Blum et al., 2000) using Attractene Transfection Reagent (QIAGEN) for 8 hr. Culture media was replenished with fresh media. After 15 hr, supernatants were collected and cells lysed in PBS 0.5% Triton X-100. Supernatants were centrifuged in order to remove cell debris. GFP levels in cell lysates and supernatants were measured by a solid-phase sandwich ELISA (Blagoveshchenskaya et al., 2002). Briefly, polystyrene 96-well plates were coated overnight with 200 μl/well of PBS plus sheep anti-GFP 1:50,000 for 1 hr at room temperature. The antibody solution was removed and the plates were then incubated for 1 hr at room temperature to block nonspecific binding by using 300 μl/well of TEB (1% Triton X-100, 0.2% gelatin, 1 mM EDTA in PBS). The TEB was removed, each well was filled with 200 μL of samples or standard curve in TBE, and the plates were incubated while shaking for 1 hr. After extensive washing, plates were incubated with 200 μL TBE plus Rabbit anti-GFP 1:20,000 for 1 hr with shaking. Next, plates were washed and incubated with 200 μl/well of Goat anti-Rabbit HRP 1:3,000 in TBE for 1 hr plus shaking. Plates were washed three times in TBE and 3 times in PBS and using a standard o-phenylenediamine assay. Percentage of secreted GFP was calculated with respect to total GFP produced (supernatant plus lysates).

Western Blotting

Equal number of cells were seeded and cultured for 24 hours. Cells were washed with cold PBS and lysed using Laemmli buffer (BioRad). All lysates were sonicated, heated for 10 min at 95°C and treated with 143 μM β-mercaptoethanol. Electrophoresis gels were loaded with the same quantity of lysates and run for 45 min at 130 V. Transfer to PDVF membranes were carried out at 65 V for 2 hr. Membranes were blocked for 2 hr at room temperature with 5% skim milk powder (Sigma-Aldrich), 2% BSA in PBS and stained with primary antibodies overnight at 4°C in 1:5 diluted blocking buffer. The next day, membranes were thoroughly washed in PBS 0.05% Tween 20 and incubated with HRP-conjugated secondary antibodies 1:5000 in 1:5 diluted blocking buffer.

Quantitative RT-PCR analysis for Phldb2 (LL5β) messenger RNA

cDNA was generated from RNA samples using the SuperScript IV Reverse Transcriptase kit (Thermo Fisher Scientific), following manufacturer's instructions. Quantitative PCRs were run using the MESA Blue qPCR Mastermix (SYBR Assay). We used specific primers for detection of *Phldb2* mRNA transcripts 1 and 2 (PrimerBank ID 23510303a1): Forward Primer AGCCGCGTTTC TGAAAGCA (1653-1671); Reverse Primer CATCCGGGCGTCTTCCATT (1773-1755). Detection of GAPDH mRNA was used for normalization.

Three-dimensional cell culture

FRCs were plated in 24-well MatTek plates at 3.5x10^3 cell/cm^2. Matured BMDCs were harvested and 150,000 cells were seeded per well in 150 μl collagen/matrigel matrix plus 20 ng/ml rmGM-CSF (Acton et al., 2012; Calvo et al., 2013; Gaggioli et al., 2007). FRC:BMDC ratio 1:43. Gels were set at 37°C for 30 min. After 24 hr, cells were fixed, permeabilized and stained for the stated cellular components.

Immunostaining of cells *in vitro*

Cells were plated on 13 mm coverslips. Cells were fixed for 15 min in 3.6% formaldehyde and permeabilized with Triton X-100 0.3% for 15 min, at room temperature. Cells were blocked with PBS 2% BSA for 1 hr at room temperature, followed by overnight incubation with corresponding primary antibodies in PBS 1% BSA. After washing, cells were incubated with Alexa Fluor-conjugated secondary antibodies plus Hoechst and/or phalloidin to reveal DNA in cell nuclei and F-actin respectively, all in PBS 1% BSA for 2 hr at room temperature, washed and mounted on glass slides for imaging. Samples were imaged in Leica TCS SP5 and SP8 STED 3X Confocal Microscopes using 63X HCX PL APO lenses or HC PL APO CS2 /1.4 63x oil lenses.

LL5β silencing by siRNA

WT FRC cell lines were transfected with four different siRNAs targeting LL5β expression (Dharmacon, GE Healthcare) using lipofectamine 2000 (Thermo Fisher Scientific). After 24 hr transfection, cells were washed and cultured in fresh media for an additional 12 hours before silencing efficiency was determined by qPCR. The following two siRNAs were selected for further assays: #1 GCAG AGUAUCAGCGGAACA and #2 GAACAAUGAAGGACCGAGA. Scrambled RNA and non-template controls were used for comparison.

Flow cytometry of LNs

Inguinal LNs were carefully dissected and digested using collagenase P at 200 μg/ml (Sigma-Aldrich), dispase II 800 μg/ml (thermos fisher scientific) and DNase I 100 μg/ml (Sigma-Aldrich) in RPMI at 37°C in a water bath. Every 10 min LNs were mixed by pipetting up and down and half of the digestion media replenished by fresh until all tissue was digested. Cell suspensions were centrifuged, resuspended in FACS buffer (PBS 2% FBS 10mM EDTA) and filtered through a 70 μm cell strainer (Corning). Cells were counted and approximately 1x10^6 cells were used for immunofluorescence staining. In brief, cells were resuspended in 100 μL FACS buffer, treated with Fc blocking for 10 min on ice and incubated with the indicated antibodies for 30 min on ice. Cells were washed extensively and resuspended in 500 μL FACS buffer. Precision Count Beads (BioLegend) were used for accurate cell count. Samples were run in a Fortessa X20 flow cytometer (BD Biosciences) at the UCL Cancer Institute and analyzed using the FlowJo software (FlowJo, LLC). Live cells were gated by FSC/SSC parameters and doublets discriminated by comparing SSC-A versus SSC-H.

QUANTIFICATION AND STATISTICAL ANALYSIS

Statistical parameters including the exact value of n, what n represents, precision measures (mean/median ± SD) and statistical significance are reported in the Figures and Figure Legends.

Statistical analysis was performed using Prism 7 (GraphPad Software). For *in vivo* experiments, naive versus inflamed LNs were compared by unpaired, parametric t test, assuming that both populations had the comparable standard deviation. For *in vitro* experiments and all other multiple comparisons, ordinary one-way ANOVA followed by Tukey's multiple comparisons test was performed.

Binomial test type for PANTHER Overrepresentation Test of cellular components (RNaseq) and biological process (Phosphoproteomics) was used to analyze changes induced by CLEC-2 binding to FRCs.

DATA AND CODE AVAILABILITY

RNaseq data and phospho-proteomics data are available: UCL research data repository: https://doi.org/10.5522/04/c.4696979; PRIDE repository ID = PXD015816

Cell Reports, Volume *29*

Supplemental Information

Fibroblastic Reticular Cells Control Conduit Matrix Deposition during Lymph Node Expansion

Victor G. Martinez, Valeriya Pankova, Lukas Krasny, Tanya Singh, Spyridon Makris, Ian J. White, Agnesska C. Benjamin, Simone Dertschnig, Harry L. Horsnell, Janos Kriston-Vizi, Jemima J. Burden, Paul H. Huang, Christopher J. Tape, and Sophie E. Acton

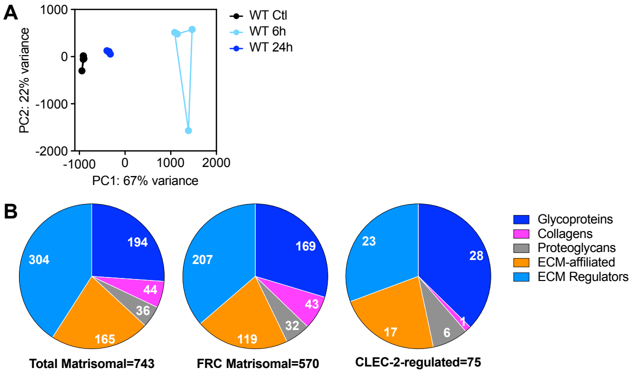

Figure S1. Matrisomal genes regulated by CLEC-2. Gene expression by RNAseq in control FRCs treated with CLEC-2-Fc for 6 and 24 hours. A) CLEC-2-Fc-regulated gene (more or equal than 2-fold) cluster in a PCA space. B) Number of genes per category of matrisomal components in the indicated datasets. Related to Figure 2.

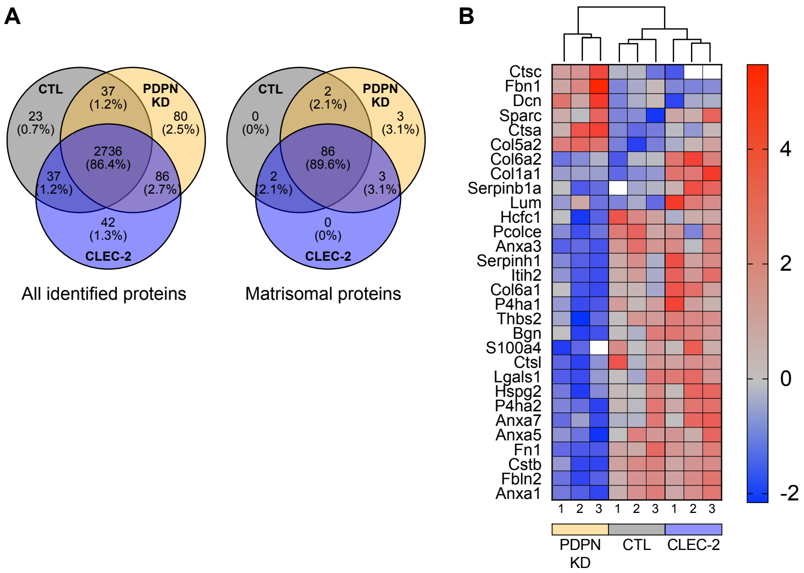

Figure S2. Proteomics of FRC-derived matrices. In vitro FRC cell line-derived matrices generated after 5 days in culture were subjected to proteomic analysis by mass spectrometry. A) Venn diagrams showing overlap between cell lines of proteins detected. Areas shown are not proportional to percentage of overlap. C) Heatmap of matrisomal proteins significantly changed by one-way ANOVA, Tukey's multiple comparisons test. Three replicates for each condition automatically clustered are shown. Colour code represents z-scores. Not detected is represented by the white squares. Related to Figure 4.

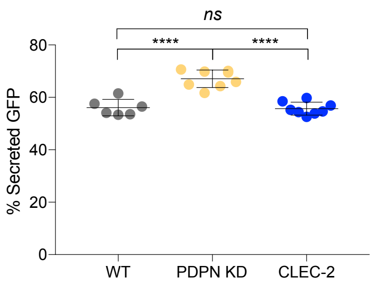

Figure S3. Secretory activity in FRC cell lines. FRC cell lines were transfected to express GFP tagged for secretion. GFP levels in cell supernatants and lysates were determined by ELISA. Dot plot shows percentage of GFP in supernatant relative to total. Dots represent replicates form independent experiments (n=2). ****P<0.00005, one-way ANOVA, Tukey's multiple comparisons test. NS, not significant. Error bars represent mean and SD. Related to Figure 4.

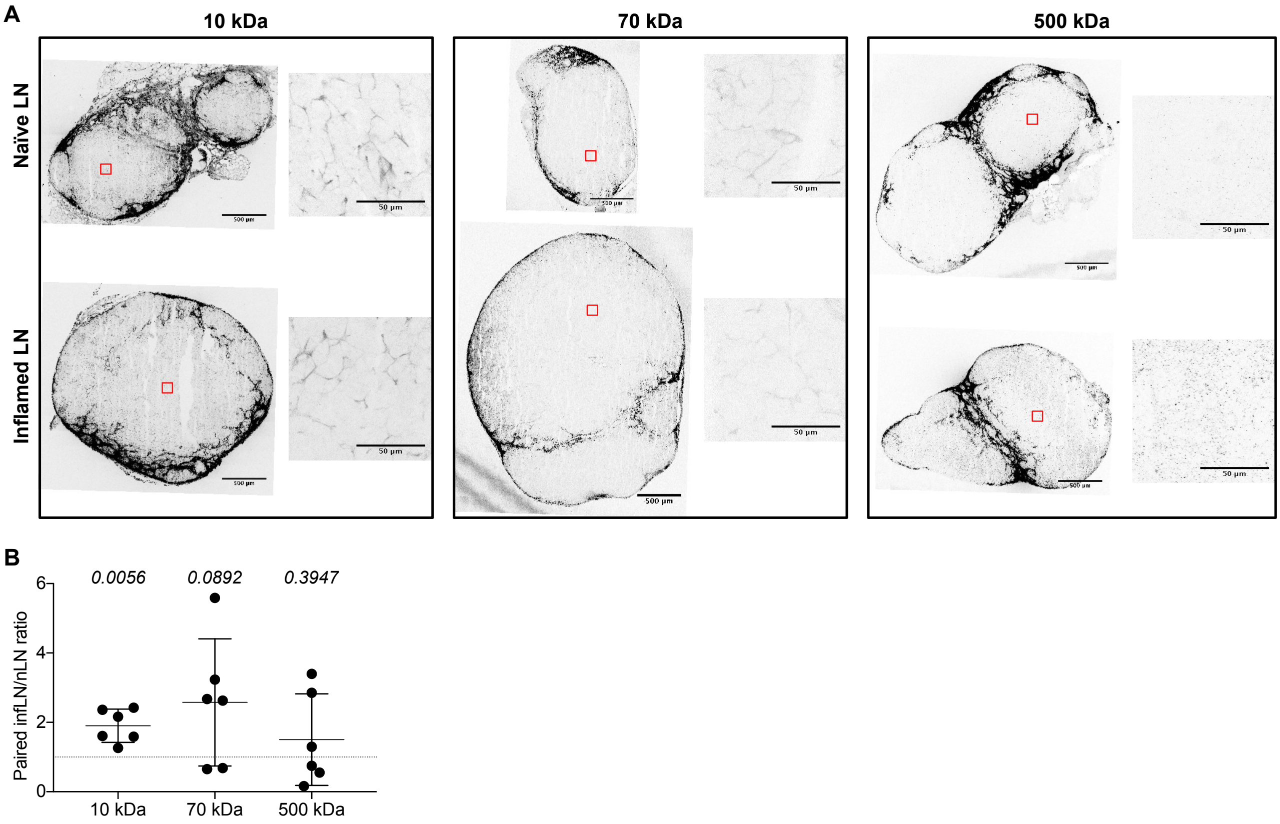

Figure S4. Antigen uptake in vivo. Mice were immunized by subcutaneous injection of IFA/OVA on the right flank. 5 days later, fluorescently labelled dextrans with the indicated sizes were injected on both flanks. A) Immunofluorescence of 20 microns thick cryosections of naïve and inflamed draining LNs 30 minutes post dextran injection. Maximum Z stack projections are shown of representative tile scans and zoomed areas are shown. B) Number of dextran-positive cells per draining LNs 90 minutes after dextran injection was determined by flow cytometry. Dot plot shows ratios between paired inflamed and naïve LNs from same individuals (n=6). Error bars represent mean and SD. P-values are shown for each dextran, unpaired t test. Related to Figure 6.

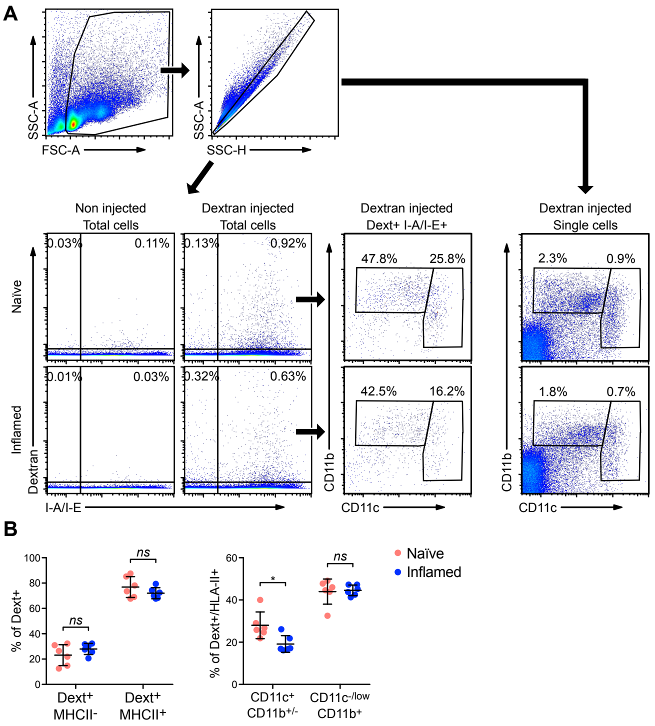

Figure S5. Gating strategy and population analysis for in vivo antigen uptake. A) Representative dot plots showing gating strategy. Live cells were gated according to FSC/SSC parameters and doublets were excluded prior to analysis. Total cells were used to gate dendritic cell (DCs) and macrophage (MF) populations. B) Percentage of the indicated cell populations within the dextran-positive subset. Each dot represents an individual mouse (n=6). Error bars represent mean and SD. *$P<0.05$, one-way ANOVA, Tukey's multiple comparisons test. Related to Figure 6.
